# Cannabinoid Receptor 2 (CB2) in Macrophages: A Promising Clinical Target for Immune Disorders

**DOI:** 10.3390/ijms26178657

**Published:** 2025-09-05

**Authors:** Hyeyoung Hailey Yoon, Natasha Lillia Grimsey

**Affiliations:** 1 Department of Pharmacology and Clinical Pharmacology, School of Medical Sciences, Faculty of Medical and Health Sciences, University of Auckland, Auckland 1023, New Zealand; hailey.yoon@auckland.ac.nz; 2 Centre for Brain Research, Faculty of Medical and Health Sciences, University of Auckland, Auckland 1023, New Zealand; 3 Maurice Wilkins Centre for Molecular Biodiscovery, Auckland 1142, New Zealand

**Keywords:** cannabinoid receptor 2 (CB2), drug development, immune disorder, immune response, inflammation, Kupffer cell, macrophage, microglia, therapeutics

## Abstract

Macrophages are essential for immune homeostasis, playing crucial roles in immune responses from initiation to resolution. They trigger acute inflammation to promote elimination of pathogens and regulate excessive immune reactions to prevent chronic inflammation and autoimmune diseases. Consequently, macrophage dysfunction contributes to the progression of many disorders that involve inflammation. Cannabinoid Receptor 2 (CB2) has emerged as a promising therapeutic target due to its role in regulating macrophage-mediated immune functions, including via modulation of cytokine secretion, migration, phagocytosis, and polarisation. CB2 activation can produce beneficial outcomes via suppressing macrophage-mediated inflammatory pathways in animal models for various diseases that involve acute or chronic central or peripheral inflammation, whereas blocking CB2 may have utility when macrophage polarisation to a “resolving” phenotype is deleterious, such as in tumour-associated macrophages. However, despite abundant promising preclinical results, the relatively few CB2-selective agonists tested in clinical trials to date have exhibited limited efficacy. Here, we provide an overview of the roles of macrophages in health and disease, thoroughly review in vitro and in vivo preclinical findings on CB2-mediated modulation of macrophage function, summarise current progress in clinical trials for CB2-targeted compounds, and discuss approaches for addressing current challenges in ongoing efforts toward developing safe and effective CB2-targeted therapeutics.

## 1. Introduction

Macrophages are specialised immune cells that play crucial roles in both the innate and adaptive immune systems. Despite being indispensable in healthy immune responses, abnormal or chronic activation of macrophages can contribute to the pathogenesis and symptomology of a wide range of disorders involving excessive inflammation. While some therapeutics targeting macrophages are available, there remains unmet clinical need which might be addressed via new therapeutic strategies and drug targets.

Cannabinoid Receptor 2 (CB2) is one such promising drug target for macrophage-related disease. CB2 expression on macrophages is regulated by inflammatory stimuli. The majority of published studies highlight CB2 agonists as promising therapeutic candidates for preventing or reversing excessive inflammation involving macrophages, though the specific outcomes can depend on the specific stimulus, disease model, and timing of treatment. Conversely, CB2 inverse agonists have also demonstrated efficacy in certain diseases, such as those associated with macrophages deleteriously acquiring a “resolving” phenotype (e.g., some cancers).

Here, we provide an overview of macrophage biology and roles in disease. We then summarise CB2 molecular pharmacology and thoroughly review in vitro and in vivo preclinical studies addressing CB2’s role in macrophage function. Finally, we summarise past and ongoing clinical evaluation of cannabinoids and CB2-selective compounds, and provide perspectives for overcoming challenges in the ongoing advancement toward successful utility of CB2 as a drug target in macrophages.

## 2. Macrophages in Health and Disease

### 2.1. General Roles and Functions of Macrophages

Macrophages primarily function as part of the body’s defence system against pathogens, acting via phagocytosis and interactions with other immune cells through antigen presentation and cytokine secretion [1,2,3].

Phagocytosis is a series of processes in which phagocytic cells, such as macrophages, recognise, engulf, and degrade large particles. Phagocytosis by macrophages not only acts as a defence against invading pathogens but also contributes to the clearance of debris and damaged tissues [4]. Macrophages recognise pathogen-associated molecular patterns (PAMPs) and danger-associated molecular patterns (DAMPs) via specific pattern recognition receptors (PRRs), such as toll-like receptors (TLRs). Once macrophages recognise PAMPs or DAMPs and then engulf these targets, they create a phagosome from the engulfed membrane and degrade the targets in the phagosome [5]. While phagocytic activity may arise as a result of macrophages encountering PAMPs or DAMPs while “patrolling” tissues via non-directional mobility, subsequent responses, such as inflammasome activation, antigen presentation and cytokine secretion, can stimulate further phagocytosis by encouraging migration and chemotactic movement of additional macrophages along a concentration gradient of chemoattractant.

Inflammasome activation is triggered by the recognition of PAMPs and DAMPs by PRRs such as NOD-like receptors (NLRs) in the cytosol, often subsequent to phagocytosis. NLR binding leads to oligomerisation with pro-caspase 1 and adaptor proteins such as ASC (Apoptosis-associated Speck-like protein containing a Caspase recruitment domain) to form a multimeric protein complex. This leads to the release of interleukin (IL)-1β and IL-18, mediated by activation of cleaved caspase-1 from pro-caspase-1 [6]. This cytokine secretion induces the production of other pro-inflammatory cytokines, such as IL-16 and tumour necrosis factor (TNF, also known as TNF-α), and consequent sequential inflammatory responses. In addition, caspase-1 can induce pyroptosis, a type of programmed cell death, forming pores in the cell membrane leading to cell swelling and rupture [7]. This releases intracellular molecules and DAMPs into the surroundings, which acts to recruit other immune cells [6].

Macrophages can also facilitate direct interactions with other adaptive immune cells through antigen presentation. This is initiated by expressing epitopes of pathogens on major histocompatibility complex (MHC) molecules on the cell surface of macrophages post-phagocytosis. Through antigen presentation, macrophages stimulate neighbouring immune cells, such as T cells or B cells, and activate adaptive immune responses, for example, T cell proliferation and antibody production by B cells [3,8].

In cases where pathogens are not eliminated completely by localised immune cells, macrophages promote a systemic inflammatory response through pro-inflammatory cytokine secretion. In particular, chemokines, a sub-category of cytokines, induce infiltration and recruitment of various other immune cells into inflammatory regions by directional chemotaxis [3,9].

Although the pro-inflammatory response is an effective way to defend the host against pathogens, it can also cause collateral damage to tissue and systemic effects, such as fever [10,11]. In a healthy individual, after eliminating pathogens, macrophages gradually reduce the pro-inflammatory response to return to immune homeostasis and simultaneously increase the anti-inflammatory response, including the production of growth factors such as vascular endothelial growth factor (VEGF), to facilitate repair of damaged tissue [12,13]. These diverse functions highlight the importance of macrophages in both defending the host and maintaining a balanced immune response [14,15].

### 2.2. Macrophage Polarisation

Macrophages are derived from two broad origins, monocyte-derived macrophages (MDMs) and embryonic tissue-resident macrophages [14,16]. Regardless of origin, macrophages possess plasticity and heterogeneity to adjust their properties in response to their environment [17]. Circulating monocytes in the bloodstream are attracted to inflammatory regions, such as infection sites, and infiltrate into damaged tissues [18]. Here, monocytes differentiate into MDMs by cytokine stimulation, which intensifies the subsequent immune response [19]. Tissue-resident macrophages acquire unique characteristics based on signals from their surrounding environment and location, for example, Kupffer cells in the liver, Langerhans cells in the skin, and microglia in the brain [16]. The plasticity of macrophages allows them to polarise toward either pro-inflammatory or anti-inflammatory effects as well as to differentiate into various phenotypes [20].

The differentiation and polarisation of macrophages are mediated by various inducers, such as hemopoietic growth factors macrophage colony-stimulating factor (M-CSF) and granulocyte–macrophage colony-stimulating factor (GM-CSF) [21]. Although the distinct function and signalling pathways induced by GM-CSF and M-CSF under various conditions are still under ongoing investigation, these cytokines play pivotal roles in monocyte maturation, differentiation and proliferation, and prolong the survival of macrophages [22,23].

GM-CSF and M-CSF elicit dose-dependent responses and differ in the downstream pathways stimulated [24,25,26]. In infectious conditions, GM-CSF levels rise due to secretion from activated leukocytes. GM-CSF acts on the heterodimeric GM-CSF receptor on monocytes and macrophages, then activates Janus-activated kinases-signal transducer and activator of transcription (JAK/STAT), mitogen-activated protein kinase (MAPK), Nuclear Factor kappa B (NF-κB), and phosphoinositide 3-kinase (PI3K) pathways to promote inflammatory responses [22,24,27,28]. On the other hand, M-CSF is constantly detectable in blood and binds to homodimer M-CSF receptors which signal through PI3K/Akt and MAPK pathways [29]. This leads to generally anti-inflammatory responses in macrophages [22,25,30,31].

Beyond stimulation and differentiation by the two types of CSFs, macrophages can be polarised into pro-inflammatory M1 (also referred to as “classically activated”) or anti-inflammatory M2 (also called “alternatively activated”) macrophages by specific triggers, such as growth factors, cytokines, or foreign pathogens [32,33]. This activation is accompanied by changes in both macrophage phenotype and function [26,34,35,36]. When circulating MDM or resting resident macrophages are activated, the cells transform from a round shape to an adherent ameboid shape, which confers advantages for crawling and infiltration through tissue barriers [18,37].

In pro-inflammatory responses, macrophages recognise PAMPs and DAMPs as well as pro-inflammatory cytokines, inducing polarisation into M1 macrophages. The most well-known example of an M1 macrophage inducer is lipopolysaccharide (LPS), derived from the external membrane of bacteria, causing inflammation and sepsis [8,9,38]. The binding of LPS to TLR4 on the macrophage surface triggers expression of M1 markers, such as CD40, CD64, CD86, and CXCL9, and the production of pro-inflammatory cytokines, such as IL-12, IL-6, TNF, and IL-1β, through the JAK/STAT, MAPK, and NF-κB pathways. Aside from foreign pathogens, interferon-γ (IFN-γ) secreted by type 1 T helper (Th1) cells also induces M1 polarisation in macrophages through the JAK/STAT1 pathways, leading to a similar pro-inflammatory response as LPS-induced M1 macrophages [39,40].

In M1 polarisation induced by both LPS and IFN-γ, macrophages undergo a metabolic shift from the tricarboxylic acid (TCA) cycle toward anaerobic glycolysis to rapidly provide an energy source for inflammation [41,42]. This metabolic shift leads to lactate accumulation and is accompanied by the production of reactive oxygen species (ROS) and nitric oxide (NO). These elements assist in eliminating pathogens but simultaneously have negative effects on cells, damaging DNA and proteins. Furthermore, sustained release of pro-inflammatory cytokines may lead to inflammatory disease due to excessive inflammation [41,43,44].

M2 macrophages maintain immune balance and avoid excessive inflammation by suppressing pro-inflammatory responses and promoting wound healing via the production of growth factors and anti-inflammatory cytokines [45,46]. M2 macrophages are classified into subtypes based on the triggers and functions, M2a, M2b, and M2c. Stimulators of M2 polarisation include IL-10, transforming growth factor-β (TGF-β), glucocorticoids (GCs), as well as IL-4 and IL-13 [23,47]. M2 macrophages typically express CD163, CD206, Arginase 1 (Arg1), and mannose receptor 1 (Mrc1), and produce anti-inflammatory cytokines, such as TGF-β and IL-10. These phenotype changes in M2 activation are mediated by peroxisome proliferator–activated receptor (PPAR)-γ and NF-κB, and JAK/STAT6 signalling [43,48]. In contrast to M1 macrophages, metabolic pathways in M2 macrophages involve the production of adenosine triphosphate (ATP) via the TCA cycle with a lower risk of DNA and protein damage than in M1 macrophages utilising anaerobic metabolism [43,44,49].

### 2.3. Dysfunction and Disruption of Macrophages in Disease

Although both M1 and M2 macrophages are essential to protect the host, disruption of the equilibrium between the types of macrophages and their activity can lead to severe disease, with chronic inflammatory disease and autoimmune disease primarily driven by abnormal pro-inflammatory responses [50,51,52,53]. Normally, macrophages naturally turn off their pro-inflammatory state after completing their pro-inflammatory response to eliminate pathogens [34]. However, persistence of this pro-inflammatory response, characterised by elevated levels of cytokines and immune cell infiltration, may result in chronic inflammation accompanied by tissue damage. Chronic inflammation involving macrophages is linked to various inflammatory diseases, including atherosclerosis and atopic dermatitis [53,54,55].

In addition, autoimmune disorders, such as inflammatory bowel disease (IBD), multiple sclerosis (MS), rheumatoid arthritis (RA), psoriasis, and systemic lupus erythematosus (SLE), occur when the immune system attacks healthy tissues instead of pathogens [30,56,57,58,59]. While abnormal function of T cells and B cells is a well-established cause of autoimmune disorders, recent studies have also highlighted the involvement of macrophages. Macrophages not only initiate and regulate adaptive immune responses but are commonly observed to infiltrate lesion sites. Interestingly, infiltrating macrophages exhibit distinct activation profiles depending on the pathophysiology of each disease. For instance, in the lesions of patients with SLE and RA, macrophages are predominantly polarised toward the M1 phenotype, leading to an excessively activated immune response. In contrast, in fibrotic diseases such as systemic sclerosis (SSc), macrophages derived from patients exhibited a notably increased M2 phenotype. Although it remains unclear whether macrophages actively drive disease progression or whether their phenotypic and functional changes are an epiphenomenon of autoimmune diseases, the differences observed in macrophages from patients or disease models compared to those from healthy controls are noteworthy [58,60].

Interestingly, IFN-γ, which induces M1 macrophage polarisation, is upregulated in several autoimmune diseases. For example, SLE and RA patients were found to have high levels of IFN-γ for a few years before being diagnosed [61,62], while MS patients exhibited increased IFN-γ in blood samples and IFN-γ-induced chemokines in lesions [63]. Continuous exposure to low concentrations of IFNs can result in macrophages becoming more sensitive to inflammatory stimuli such as TNF and TLR ligands, as well as to IFNs. This heightened sensitivity is mainly observed in lesions of autoimmune disease and is mediated by the STAT pathway [62,64].

In contrast to persistent pro-inflammatory responses observed in immune disorders, cancer involves inappropriate immune regulation and activation of wound healing pathways, which is linked to uncontrolled proliferation and angiogenesis in the tumour microenvironment [65]. To take the case of melanoma, DNA damage in skin cells from ultraviolet radiation induces infiltration of macrophages into the area to promote the removal of damaged cells. After the removal of damaged tissues, anti-inflammatory macrophages suppress pro-inflammatory responses and promote wound healing. However, as the tumour microenvironment forms, M2 macrophages can differentiate into tumour-associated macrophages (TAMs) which can secrete factors promoting tumour growth and metastasis via promoting cell migration and angiogenesis [43,65,66].

Conventional pharmacotherapies for chronic inflammatory diseases often have relatively untargeted effects on the immune system, in some cases producing overall immune suppression. Although effective in many patients, limitations include increased susceptibility to infections and malignancy which pose difficult trade-offs for long-term use and prevent use in some patients [43,67,68]. More modern approaches aim to specifically target pathogenic pathways, including those mediated by macrophages. Such drugs, a number of which are Food and Drug Administration (FDA)-approved with more currently in clinical trial [69,70], regulate immune activity by blocking or enhancing the molecular regulators or targets of macrophages including GM-CSF, CSF-1R, and CD40, aiming to modulate macrophage polarisation toward either M1 or M2 phenotypes depending on pathogenic characteristic. For instance, enhancing M1 function can promote antitumour effects or suppress fibrosis in autoimmune disorders, whereas inducing M2 macrophages may support tissue repair or suppress excessive M1-driven inflammation [60,69]. Nanotechnology-based drug delivery systems are also in development and show promise in targeting macrophages at specific sites, such as the tumour microenvironment or in the brain, to improve precision and clinical efficacy [69].

Aside from traditional pharmacological approaches, cell-based therapies are in development [69]. For example, chimeric antigen receptor–macrophage (CAR-M) therapy involves genetic reprogramming to enhance phagocytic function against tumour cells and improve antigen presentation. Another promising approach is Ixmyelocel-T, an autologous cell therapy enriched with bone marrow-derived M2-like macrophages, which has demonstrated therapeutic benefits in chronic inflammatory conditions, such as cardiovascular disease.

Despite these advances, however, macrophage-targeted and -based therapies still face challenges in producing robust therapeutic efficacy and durability. As macrophage polarisation represents a gradual, spectrum-like process rather than two distinct categories, macrophage-targeted molecules to induce or suppress polarisation may not elicit significant effects in cells exhibiting moderate levels of activation. In addition, because of the plasticity of macrophages, macrophage-based cell therapies may have difficulty in maintaining the functional phenotype of administered macrophages until they arrive the target region. Nevertheless, continued research focusing on optimising macrophage modulation and delivery strategies is expected to further expand therapeutic impact [69].

## 3. Cannabinoid Receptor 2 (CB2) Molecular Pharmacology

### 3.1. Endocannabinoid System

The endocannabinoid system regulates various physiological processes, including contributing to the maintenance of immune homeostasis. Named due to responsiveness to compounds from the *Cannabis sativa* plant, the system consists of endocannabinoids, cannabinoid receptors, and regulatory enzymes that synthesise and degrade endocannabinoids [71]. The concentration of endocannabinoids is governed by enzymatic activity through anabolic and catabolic enzymes [72,73]. The levels of circulating endocannabinoids are adjusted in response to various stimuli, such as food consumption and physical exercise. Inflammation can elevate levels of anandamide (AEA) and 2-arachidonoylglycerol (2-AG), which are the two most well-known endocannabinoids (Table 1) [74,75]. These endocannabinoids subsequently bind to cannabinoid receptors and lead to their activation [76]. For instance, brain injury-induced neuronal damage increases AEA levels, which activates cannabinoid receptors on microglial cells leading to suppression of excessive immune responses and protecting the central nervous system (CNS) tissue from further damage [77]. Similarly, in a rat spinal cord injury model, AEA levels initially increased following the injury but subsequently declined, whereas 2-AG levels exhibited a sustained elevation over the post-injury period [78,79].

The two established cannabinoid receptors, CB1 and CB2, share structural similarities that enable endocannabinoids and phytocannabinoids to bind to both receptors and activate the endocannabinoid system. In early reports of cannabinoid receptors, the function of each cannabinoid receptor was largely assumed to correlate closely with the sites of predominant expression [80]. CB1 is expressed at the highest levels in the CNS, where it modulates neurotransmitter release, such as γ-aminobutyric acid (GABA), to regulate the neurobiological processes in the brain, including memory and cognition, appetite, and sleep [81,82]. Consistent with these roles, CB1 mediates the psychoactive effects of Cannabis.

In contrast, CB2 is predominantly found in the periphery, and best recognised for expression in, and ability to modulate, the immune system [80]. Among resting immune cells, CB2 expression is generally reported to be most abundant in B cells followed by natural killer (NK) cells, monocytes, and T cells, though expression can be regulated by various stimuli (see Section 4.1) [83,84,85]. The subcellular distribution of CB2 protein also varies between cell types and potentially in response to stimuli, with considerable or predominant intracellular expression being reported in a range of cell types, including immune cells, presumably able to be activated by intracellularly synthesised ligands and lipophilic cannabinoids that are able to cross the plasma membrane [86,87,88,89,90]. A seminal study supporting the importance of CB2 in immune responses demonstrated that the immunomodulatory effects of Δ9-tetrahydrocannabinol (THC), the major psychoactive compound in *Cannabis*, were lost in CB2 knockout (KO) mice, while the cannabinoid central nervous system effects remained. Specifically, THC inhibited T cell activation via macrophages only in the presence of CB2, with no effect observed in CB2 KO mice. This demonstrated the possibility of CB2-targeted therapy for immunomodulation without the induction of psychoactivity [91].

More recent research has challenged the initially adopted simple functional boundaries between CB receptors, leading to ongoing debate and unresolved implications. There is evidence indicating the presence and activity of CB1 in the immune system, such as T cells, B cells, and monocytes, as well as in peripheral organs like the liver and pancreas [92,93,94,95,96]. In parallel, CB2 has also been discovered in the CNS, including microglia, oligodendrocytes, astrocytes, and neurons [72,97,98]. Many studies have observed increased expression of CB2 in the CNS under neuroinflammatory conditions, raising the possibility of targeting CB2 for immunomodulation in the CNS [95,99]. Although both CB1 and CB2 have immunomodulatory properties, CB2 has emerged as a more promising drug target for managing inflammatory responses both due to greater expression and potency of effects in most immunoregulatory contexts and, importantly, because targeting CB2 avoids the psychoactive effects associated with CB1 activation [80,82].

In addition to the main cannabinoid receptors, CB1 and CB2, other GPCRs have also been proposed as putative cannabinoid receptors, GPR18, GPR55, and GPR119 [100,101,102]. Although still classed as orphan receptors due to lack of consensus regarding their endogenous ligand(s), all have been reported to respond to some cannabinoid ligands and are potential drug targets, with both GPR18 and GPR55 attracting attention as potential targets for immunotherapy along with CB2 [103,104,105,106]. CB2 has also been suggested to form functional heteroreceptor complexes with CB1, GPR18, and GPR55, as well as other non-cannabinoid receptors, further adding complexity to the potential for CB2-mediated responses and cross-regulation [107]. For example, CB1-GPR18 dimerisation in microglia produced distinct signalling properties such as negative crosstalk and cross-antagonism. These heteromers were upregulated upon microglial activation and were also detected in Alzheimer’s disease (AD) model mice, suggesting their potential involvement in neuroinflammatory processes and neurodegeneration [108]. Some cannabinoids also interact with other non-GPCR effectors, including transient receptor potential vanilloid 1 (TRPV1) channel and peroxisome proliferator–activated receptors (PPARs) [104,109,110]. The potential for overlapping pharmacology and interactions between these receptors is important to consider in the design and interpretation of studies involving cannabinoids and drug development for these targets.

### 3.2. Exogenous Cannabinoids

Exogenous cannabinoids are acquired through two main approaches: they can either be naturally derived from the *Cannabis* plant (phytocannabinoids) or chemically synthesised.

Since ancient times, the *Cannabis* plant has been used for a wide range of indications, from recreational to ritualistic to medicinal [82,111]. Traditional medicinal use has included treating pain, nausea, and psychological illnesses like anxiety. Of hundreds of potentially bioactive compounds in *Cannabis*, some of the best studied are THC and cannabidiol (CBD) (Table 1) [112].

Synthetic cannabinoids are designed to replicate, or hone, the pharmacological properties of natural cannabinoids. Compounds like CP55,940 and WIN55,212-2 are synthetic cannabinoid ligands commonly used in pharmacological studies that activate both CB1 and CB2. Achieving selectivity between CB1 and CB2 (and other potential targets; see Section 3.1 and Table 1) poses both a challenge and opportunity for utilising synthetic cannabinoids therapeutically to avoid potential adverse effects, such as preventing psychoactivity via CB1 activation when a ligand is intended to be targeted to CB2 receptors in inflammatory diseases [113,114,115].

To address this selectivity issue, there has been steady development of CB2-selective agonists and antagonists via screening for new chemical scaffolds and making subtle structural modifications to the lead molecules. Examples of agonists with improved selectivity toward CB2 include JWH-133 and HU308, and inverse agonists AM630 and SR144528 (SR2). A range of such compounds have been utilised in research on the roles of CB2 in macrophage function (Table 1). More recent developments include efforts toward physicochemical optimisation of CB2 ligands with oral bioavailability in mind, and the availability of CB2 crystal and cryo-EM structures is expected to further accelerate novel ligand development [113].

**Table 1:** CB2-selective and non-selective agonists and inverse agonists utilised in studies on the role of CB2 in macrophages.

Compound Name(s)	Description (Compounds Are Synthetic, Unless Indicated)	CB2 Affinity ^▲^ (or Functional Potency ^◆^)	CB2 > CB1 Binding ^■^ (or Functional ^◆^) Fold Selectivity
Anandamide (AEA)	Endocannabinoid non-selective partial agonist	250 nM	0.6 °
2-Arachidonoylglycerol (2-AG)	Endocannabinoid non-selective agonist	400 nM	2.0 °
Δ9-Tetrahydrocannabinol (THC)	Cannabis-derived non-selective partial agonist	20 nM	0.7 °
Cannabidiol (CBD)	Cannabis-derived non-selective context-dependent (allosteric? [116]) partial agonist or inverse agonist	3770 nM [117]	1.8 ° [117]
AM1241 (Racemic mix [Rac.] vs. R/S enantiomer often unspecified)	CB2-selective partial (protean? [118]) agonist	Rac. 10 nM [R] 7.9 nM [S] 250 nM [118]	Rac. 32–350 [R] 38–330 [S] >15–150 ° [119,120]
β-(E/trans)-Caryophyllene (BCP)	CB2-selective agonist	160 nM [121]	>63 [121]
CP55,940	Non-selective agonist	2.0 nM	1.5 °
GP1a	CB2-selective agonist	0.037 nM ^m^ [122]	9800 ^m^ [122]
GW405833	CB2-selective partial agonist	3.9 nM [123]	1200 [123]
GW833972A	CB2-selective agonist	50 nM ^◆^ [124]	630 ^◆^ [124]
HU308	CB2-selective agonist	32 nM	>360
JWH-015	Slightly CB2-selective agonist	13 nM	28 °
JWH-133	CB2-selective agonist	16 nM	130 °
MDA7 (NTRX-07) (racemic mix)	Slightly CB2-selective agonist	500 nM [125]	>24 [125]
NESS400 (GP2a)	Slightly CB2-selective agonist	7.6 nM ^m^ [122]	73 ^m^ [122]
O-1966 (0-1966[-A])	CB2-selective agonist	23 nM [126]	220 [126].
O-2137 (racemic mix of O-1966 and O-1967)	CB2-selective agonist	10 nM [127]	240 [127]
WIN55,212-2	Non-selective agonist	1.3 nM	8.9 °
AM630	CB2-selective (protean? [128]) inverse agonist	32 nM	120 °
SMM-189	Slightly CB2-selective (non-competitive? [129]) inverse agonist	120 nM [129]	39 [129]
SR144528 (SR2)	CB2-selective inverse agonist	7.9 nM	>960

^▲^ CB2 affinity (Ki) (rounded to 2 sf), except ^◆^ EC_50_ based on functional assay. Values from a review aggregating data from multiple studies (converted from pKi) [113] or as indicated, and for human CB2 unless indicated ^m^. ^■^ Fold selectivity for CB2 > CB1 (rounded, 2 sf), based on binding affinity, except ^◆^ based on functional potency. Values from a review aggregating data from multiple studies [113] or as indicated, and for human receptors unless indicated ^m^. ° Indicated compounds have reported activity at targets other than CB1 and CB2, such as GPR18, GPR55, TRPV1, and PPARs [104,109,110]. Some compounds may not have been tested for affinity/efficacy at targets other than CB2 and CB1. ^m^ Data from mouse receptors.

As well as for research purposes, cannabinoids have drawn attention from the public and researchers for their potential clinical efficacy, and the use of selected cannabinoid ligands, to date primarily those directly derived from *Cannabis*, has been approved in many Western countries [112,130]. Examples of approved cannabis-derived medicines, and clinical trials for CB2-selective compounds, will be discussed in Section 7 (Table 2 and Table 3).

### 3.3. Cannabinoid Receptor 2 (CB2) Activation and Signalling

The cannabinoid receptors belong to the G protein-coupled receptor (GPCR) family. GPCRs have seven transmembrane domains, forming extracellular domains contributing to the recognition of ligands and intracellular domains for signal transduction, with the transmembrane domains facilitating both aspects. Intracellular signal transduction involves heterotrimeric G proteins, consisting of Gα and Gβ/γ subunits. Agonist binding to the receptor triggers signal transduction via association of a G protein heterotrimer with the receptor and exchange of guanosine diphosphate (GDP) for guanosine triphosphate (GTP) on the Gα subunit. With GTP bound, the Gα subunit dissociates from the Gβ/γ subunits, and both can then activate downstream pathways by interacting with enzymes such as adenylate cyclase and phospholipase C, ion channels, and other effectors. In terminating the signal, GTP bound to the Gα subunit undergoes hydrolysis to GDP, after which the Gα subunit reunites with Gβ/γ ready for potential re-activation. Even in the continued presence of receptor ligand, responses typically reduce over time due to the GPCR undergoing desensitisation and internalisation. Desensitisation initiates when GPCR kinases (GRKs) phosphorylate the receptor, which induces the binding of β-arrestin to the G protein leading to receptor internalisation [131].

After CB2 activation, different subtypes of Gα proteins, such as Gαi, Gαs, and Gαq, can be activated and stimulate various downstream pathways. By far, the vast majority of studies on CB2 signalling have reported predominant Gαi coupling, activation of which leads to inhibition of adenylate cyclase and consequent reduced cyclic adenosine monophosphate (cAMP) synthesis. However, two studies stimulating immune cells with CB2-selective ligands JWH-133 (thrombin-induced rat primary microglia) and HU308 (human primary peripheral blood mononuclear cells; PBMC) indicated the potential for Gαs coupling in specific contexts [132,133]. Activation of the Gαs subunit has opposite effects to Gαi on cAMP by stimulating adenylate cyclase [134,135]. In addition, Gαq-mediated signalling by CB2 activation can reportedly be induced by intracellular rather than cell surface CB2 [136]. Intracellular administration of CB2 agonists led to Gαq-mediated upregulation of Ca^2+^ signalling, while extracellular administration of CB2 agonists was ineffective. This finding, along with earlier-noted studies indicating intracellular CB2 expression in a range of cell types (Section 3.1), suggests that the location of the receptor should also be considered when studying CB2 receptor signalling, expanding the scope of pharmacological studies on CB2 agonists and considerations in therapeutic design [88,113,136]. Through the pathways involving Gα and Gβ/γ subunits, CB2 activation modulates various cellular functions via secondary messengers, such as adenylate cyclase, potassium channels, MAPKs (e.g., extracellular signal-regulated kinase [ERK], p38), and PI3K/Akt [132,135]. After initial activation, CB2 can interact with β-arrestins and induce downstream signalling pathways, typically inducing ERK activation [137,138,139]. Subsequent to arrestin interaction, CB2 internalises and can recycle back to the cell surface [140].

Interestingly, single nucleotide polymorphisms (SNPs) in the CB2 gene, some of which are non-synonymous (alter the amino acid coding sequence) and occur at high frequency in human populations overall, have been suggested to impact CB2 expression and signalling and have been linked with the incidence of immune-related disorders [141]. CB2 is also notable for relatively large disparity the between human and rodent amino acid sequences, which can also impact consistency of ligand engagement and/or downstream signalling between species [118].

### 3.4. Beyond Canonical Signalling: Biased Signalling and Allosteric Modulators

Over recent years, it has been increasingly appreciated that considering GPCR activity as a simple linear signalling model is not sufficient to explain the full spectrum of possible signalling outcomes from activation. Ligand binding to a GPCR, an allosteric protein, induces a tertiary structural modification. This alteration in the receptor structure can vary between ligands and modifies the binding sites for downstream associated proteins, such as G-proteins or β-arrestins. This concept, known as biased agonism or functional selectivity, can result in different ligands acting at a GPCR being able to produce differing patterns of signalling. As an example, contrary to the conventional concept of only contributing to receptor desensitisation, β-arrestin has been shown to induce intracellular signalling cascades independently of G proteins. This unique aspect of GPCR activation has attracted significant attention due to the potential for different ligands acting at the same GPCR to be able to produce varied outcomes based on the tendency to more strongly induce G protein (or particular G protein subtype) versus arrestin signalling [142,143,144,145]. Attributes of biased agonism are thought to be utilisable to maximise therapeutic effects and minimise adverse effects and, if so, would be invaluable to consider in the development of new drugs targeting GPCRs [143,146].

Biased agonism at CB2 has indeed been demonstrated, including the potential to distinguish G protein-mediated from arrestin-mediated signalling pathways [120,134,147,148]. Some differences in bias patterns between human and rodent CB2 have been indicated, which might have important consequences for translatability of preclinical models [118,120,134]. Only a few studies have investigated the effects of biased CB2 agonists in vivo. LY2828360, a G protein-biased CB2 agonist, suppressed cAMP accumulation and enhance ERK1/2 signalling with differing temporal patterns from CP55,940, without recruiting β-arrestin in vitro [149]. In vivo, administering LY2828360 along with an opioid agonist was reported to attenuate neuropathic pain and opioid dependence without developing tolerance, which was CB2-dependent based on comparison to KO mice. However, there was not a direct comparison to an alternative CB2 agonist, implying the impact of bias and/or temporal signalling profile could not be concluded. A direct comparison between in vivo effects of two agonists thought to have opposing biases, JWH-133 as cAMP/G protein-biased and GW833972A as arrestin-biased [134], has been undertaken for a chemically induced rat model of osteoarthritis [150]. JWH-133 produced sustained analgesia, whereas GW833972A rapidly lost efficacy which may have related to the increased propensity for arrestin interaction with this ligand. However, much more remains to be learned about the potential opportunities and consequences from CB2-mediated biased agonism.

Further extensions to GPCR signalling complexity include subcellular location bias (see Section 3.3) and temporal signal integration [151,152,153], both of which have been suggested to be relevant for CB2 signalling and could impact on therapeutic design and outcomes. Moreover, dimerisation (Section 3.1) and/or signalling crosstalk from co-stimulation or ligand polypharmacology activating different targets in the same cells will also impact signalling and downstream functional outcomes.

Allosteric modulators offer an attractive approach to modifying GPCR activity by tuning endogenous agonist responses up or down, which can hold advantages for avoiding adverse effects associated with direct receptor activation/blockade by exogenous compounds [154]. While well developed for some GPCR classes, and some well-established allosteric modulators exist for CB1 [155], few modulators are currently available for CB2. To date, CB2 allosteric modulators include Ec2la (C2) and CBd-DMH, but have undergone only limited characterisation and are not necessarily specific for CB2 [156,157,158]. A recent structural description of a putative CB2 allosteric binding site may assist in accelerating discovery and development of further CB2 allosteric ligands [159]. Interestingly, Ec2la impacts on CB2 signalling can differ depending on the orthosteric ligand applied, indicating further complexity for consideration in developing allosteric modulators for therapeutic use, but additional opportunity for fine-tuning responses via this receptor [159].

## 4. Regulation of CB2 Expression in Macrophages During Immune Responses

CB2 expression is regulated during immune responses, with the level associated with the activation state of immune cells and the presence of immune-related diseases. Studies investigating the modulation of CB2 expression in macrophages during immune responses have yielded varying results depending on the types of macrophages used, such as microglia, Kupffer cells, and peritoneal macrophages, and the type of stimulation including LPS, IFN-γ, and phorbol-12-myristate-13-acetate (PMA).

Such stimuli have been applied widely in vitro to differentiate/polarise cell lines mimicking monocyte or macrophage phenotypes. Popular cell line models include BV-2 (mouse, microglia), HL-60 (human, promyelocytic leukaemia monocyte-like line from which CB2 was first cloned) [160], J774 (mouse, macrophage), N9 (mouse, microglia), RAW 264.7 (mouse, monocyte/macrophage), spontaneously immortalised microglia (SIM)-A9 (mouse), THP-1 (human, monocytic leukaemia), and U937 (human, monocytic leukaemia). While cell lines have advantages in practicality, fidelity in faithfully modelling native cells is debated. Use of primary cells, either by harvesting monocytes and differentiating/polarising ex vivo, or harvesting macrophages after stimulating an immune response in vivo, provide models with greater likelihood of accurately representing in vivo phenotypes. Animal models of disease provide more complex models mimicking interactions between inflammatory pathways, though with varying degrees of translatability to human disease. Ex vivo human primary cells or tissue from donors with disorders of interest are extremely valuable in the study of human disease.

### 4.1. Regulation of CB2 Expression in Response to Inflammatory Stimuli

Phorbol 12-myristate 13-acetate (PMA), an inducer of differentiation from monocytes to macrophages, has been reported to attenuate CB2 expression in human monocytes. In THP-1 cells differentiated with 10 nM PMA for 48 h, CB2 gene expression decreased by more than 80%, whereas CB1 expression increased by approximately 8-fold [92]. After differentiation of human primary monocytes with 10 nM PMA for 5 days, CB1 mRNA expression increased by 2- to 3-fold compared to undifferentiated monocytes, whereas CB2 expression was reduced to less than half. Despite this reduction, CB2 expression levels remained more than three times that of CB1 expression [92]. The downregulation of CB2 protein expression following PMA stimulation was similarly observed in HL-60 cells by radioligand binding assay [161].

Inflammatory stimuli, such as LPS and IFN-γ, have been shown to induce macrophage activation while simultaneously modulating CB2 expression, with either upregulation or downregulation depending on the experimental condition.

IFN-γ-induced upregulation of CB2 mRNA and protein expression was observed in various types of macrophages, including BV-2 microglia, murine peritoneal macrophages, and primary cultured murine microglia [102,162,163]. Another representative stimulus for pro-inflammatory response in macrophages, LPS, showed a similar pattern of CB2 expression in RAW264.7 cells and rodent primary microglia [164,165]. However, in parallel, LPS stimulation was also shown to suppress CB2 expression in rat microglial cells, mouse peritoneal macrophages, and BV-2 cells [163,166,167,168]. CB2 mRNA and protein expression were assessed in LPS-stimulated U937 cells, which exhibited notably reduced levels of both mRNA and protein [169]. A similar regulatory pattern was observed for GPR55, a receptor that binds cannabinoid ligands and exert immunomodulatory functions (see also Section 3.1). In both mouse primary microglia and BV-2 cells, LPS stimulation suppressed GPR55 expression, while IFN-γ stimulation slightly reduced expression in primary microglia but increased it in BV-2 cells [102].

CB2 expression can also vary with the duration of the inflammatory stimulus. Changes in CB2 gene expression followed opposite patterns in response to two inflammatory stimuli, LPS and IFN-γ, with the expression either increasing before decreasing or decreasing before increasing within a 24 h period [102,166,170]. In BV-2 cells treated with LPS (100 ng/mL), CB2 gene expression decreased to approximately half of control mRNA within 1 h, reaching a minimum at 2 h, and began recovering from 4 h onwards. However, mRNA levels remained roughly half of the baseline even at 24 h [170]. In concentration-response experiments using 1, 10, and 100 ng/mL of LPS, higher concentrations induced greater suppression of CB2 gene expression, although expression levels at 4 h and 8 h were similar [102]. Analogous patterns were observed in rodent primary microglia, where treatment with LPS (100 ng/mL) for 18 h [102] or LPS (50 ng/mL) for 6 h resulted in decreased CB2 gene expression, which recovered by 24 h [166]. In vivo, systemic administration of LPS (5 mg/kg, intraperitoneally [i.p.]) in mice led to a marked reduction in microglial CB2 expression at 3 h post-injection, followed by an increase above baseline levels at 24 h [170].

In contrast, treatment with IFN-γ in BV-2 cells produced upregulation of CB2 mRNA, with concentration–response studies using 50, 100, and 200 U/mL producing a more pronounced upregulation at higher concentrations after 8 h, with further upregulation after 18 h [102]. Similar findings for acute with IFN-γ (100 ng/mL) treatment in BV-2 cells were later reported, where CB2 gene expression was increased after 3 h, reaching approximately a 2-fold elevation at 8 h. However, with longer treatment (24 h), expression declined to about half of baseline [170]. In rodent primary microglia, treatment with IFN-γ (200 U/mL) for 18 h slightly reduced CB2 expression (to ~80% of control), although the magnitude of suppression was considerably less than that induced by LPS [102]. In another microglial cell line, SIM-A9, a marked reduction in CB1 and CB2 mRNA expression was observed after treatment with LPS (1 µg/mL) and IFN-γ (20 ng/mL) for 24 h [171].

Mouse primary microglia differentiated with GM-CSF, IFN-γ, or M-CSF treatment for 24 h alone did not exhibit significant alteration in CB2 mRNA expression, but both IFN-γ and GM-CSF added together, a combination associated with induction of M1 polarisation, produced approximately 10-fold upregulation [167]. CB2 expression may also be upregulated under conditions inducing M2 polarisation [166]; these findings are detailed in Section 5.5.

Inflammatory disease-related stressors have been shown to upregulate CB2 expression. Oxidised LDL (oxLDL), which is associated with the development of atherosclerosis, increased CB2 expression in rat primary peritoneal macrophages and RAW264.7 cells [172], and hypoxia elevated CB2 gene expression and the level of immunohistological staining in mouse bone marrow-derived macrophages [173]. In contrast to inflammatory stressors, minocycline can be used as a treatment for neuroinflammation. Nevertheless, minocycline treatment of microglia from a collagenase-induced germinal matrix haemorrhage rat model led to increased CB2 mRNA and protein expression [174].

The mechanism of CB2 transcriptional regulation was investigated in mouse primary microglia and immortalised microglia. Transcription factor nuclear factor erythroid 2-related factor (NRF2), which is upregulated under oxidative stress and inflammation and is a recognised coordinator of anti-inflammatory effects, was associated with CB2 mRNA induction [175].

### 4.2. Regulation of CB2 Expression in Inflammatory Diseases

Research comparing CB2 expression levels in healthy individuals to those with specific inflammatory conditions (and associated animal models) has also highlighted the modulation of CB2 receptor expression during immune responses.

In multiple sclerosis (MS), a chronic autoimmune inflammatory disease, upregulation of CB2 expression in microglia compared to those of healthy control has been observed in both human post-mortem tissue and animal models. In human brain and spinal cord, increased abundance of CB2-positive microglial cells was associated specifically with lesions, though with different cell distribution between acutely active and chronic plaques [176,177,178]. In experimental autoimmune encephalomyelitis (EAE) mouse models, which are commonly utilised to mimic MS, a remarkable increase in CB2 expression in the CNS was observed. Furthermore, there was a significant elevation of CB2 expression in activated microglia and peripheral monocytes compared to their resting state [167,179]. Similar upregulation of CB2 mRNA and/or increased abundance of CB2-positive microglia has been observed in the Theiler’s murine encephalomyelitis virus (TMEV) MS model [180], though the upregulation was transient. Also in the TMEV model, CB2 expression correlated positively with pro-inflammatory cytokine levels, as well as with concentrations of 2-AG and palmitoylethanolamide (PEA, a lipid mediator suggested to be able to interact with the endocannabinoid system) [180]. In a study on human primary PBMC, CB2 (and CB1) mRNA expression was induced by TNF in cells from healthy subjects, and CB2 expression was higher in unstimulated blood from MS patients than that from healthy subjects [93].

Amyotrophic lateral sclerosis (ALS) is another neurodegenerative disorder in which progression involves activated microglia and macrophages. CB2 expression was associated with and upregulated in microglia and macrophages in spinal cords from ALS patients [176]. Huntington’s disease (HD) is a neurodegenerative disorder caused by a genetic mutation in the huntingtin protein, resulting in progressive deterioration of motor and cognitive functions. Increased microglial CB2 expression was observed in both HD patients and mouse models (see also Section 6.3) [181].

Ischaemia, although itself is not an immune disease, triggers an immune response due to nerve and tissue damage. DAMPs originating from the injured tissue initiate an innate immune response that leads to polarisation of microglia into phagocytic microglia and the release of pro-inflammatory factors. Although essential for recovery, this immune response following an ischaemic stroke can sometimes lead to adverse outcomes, such as cytokine storm [182]. Therefore, researchers studying ischaemia emphasise the importance of understanding the post-ischaemia immunomodulatory response [183,184]. Several studies have reported upregulation of CB2 expression in circulating monocytes or microglia under hypoxic ischaemia or ischaemic stroke conditions [185,186,187,188]. Interestingly, elevation of microRNA miR-665, a putative regulator of CB2 expression, was also found in monocytes from patients after suffering acute ischaemic stroke in comparison with healthy controls [185].

Increased CB2 expression is also observed in human immunodeficiency virus-1 (HIV-1) infected human primary macrophages [189], as well as in microglia from human brain tissue of patients with neuroinflammatory disorders, such as PD [190] and AD [191,192]. Accordingly, CB2-targeted PET ligands have been proposed as a potential approach for detecting neuroinflammation in living patients. [^11^C]NE40 reported increased CNS CB2 expression in mice with elevated CB2 from artificial expression [193] and a mouse model of neurodegeneration [194], though there was no change in binding in a rat stroke model (despite CB2 expression being subtly increased and correlated with a macrophage/microglia marker, based on immunohistochemistry) [195]. Increased binding of an alternative CB2 PET tracer, [^11^C]A-836339, was also detected in the brains of mice with neuroinflammation induced by LPS and amyloid plaque deposition [196], though a later study indicated this tracer lacked sensitivity. In a small human trial, a reduction in [^11^C]NE40 binding was observed in AD patients [197]. This finding was in contrast to those typical from animal model and post-mortem studies, and it is not resolved whether the unexpected binding patterns of [^11^C]NE40 were genuine difference in CB2 expression patterns, relate to low sensitivity or selectivity of the tracer, or could indicate a disease-associated change in affinity state or degree of endocannabinoid tone/competition that might impact [^11^C]NE40 binding.

On the other hand, some post-mortem human tissue studies have reported that CB2 expression was inhibited under inflammatory conditions. For instance, CB2 expression was reduced in macrophages from patients with IBD [198]. In a study investigating the reasons for increased susceptibility to spontaneous bacterial peritonitis (SBP) in cirrhotic patients, a condition marked by severe liver damage due to chronic inflammation [199], researchers identified suppression of CB2 expression in monocytes from cirrhotic patients compared to healthy subjects [169]. At the cellular level, this suppression was more pronounced in cirrhotic patients with co-existing SBP, affecting both mRNA and protein levels of CB2 in circulating monocytes and peritoneal macrophages.

Considering the overall findings of studies to date, while it is well known that CB2 expression is modulated by immune response and inflammatory disease, the result and tendency of the regulation of CB2 expression are without consensus and seem highly context dependent.

## 5. CB2-Mediated Modulation of Inflammatory Response in Macrophages

Activation of CB2 in macrophages by agonists, such as 2-AG, AEA, and exogenous cannabinoids, is associated with immunomodulatory effects via macrophages, including alteration of phagocytosis, migration, and cytokine production (introduced in Section 2.1). Although the majority of studies have demonstrated that CB2 activation promotes anti-inflammatory responses and inhibits inflammation, the specific outcomes of CB2 activation can differ depending on the origin of the macrophages, type of immune stimulus, and stage of progression of the immune response.

### 5.1. Phagocytosis and Antigen Presentation

Both endogenous and exogenous cannabinoids have been shown to modulate the phagocytic function of macrophages. Acute (30–60 min) incubation with 2-AG (1–5 μM) stimulated dectin-1-mediated phagocytosis of zymosan in mouse peritoneal and alveolar macrophages, while the phagocytosis of other targets, such as latex beads, *Escherichia coli*, Staphylococcus aureus, and apoptotic cells, were unaffected [200]. The enhanced zymosan phagocytosis was indicated to require CB2 activation (due to loss of effect when selective inverse agonist SR2 was co-incubated) and the PI3K pathway [200]. Similar findings were reported in dihydroxy-vitamin D3-differentiated HL-60 cells treated with 2-AG (≥10 nM), where enhanced phagocytosis of opsonised zymosan. The effect of 2-AG on phagocytosis was reversed by SR2 and involved Gαi/o proteins, PI3K and ERK [201].

Efferocytosis aims to resolve inflammation and restore tissue homeostasis by recognising and clearing apoptotic cells by macrophages. In human primary MDMs, endocannabinoid AEA (100 nM) significantly increased efferocytosis in a CB2- and GPR18-dependent manner, as supported by the inhibitory effects of their respective antagonists (SR2 and O-1918 for GPR18) [106]. Consistent with findings that CB2 activation can enhance macrophage phagocytosis, CB2 KO significantly reduced phagocytic activity of both M0 and TGF-β-induced M2c mouse microglia, compared to equivalently stimulated microglia from wild-type mice. However, phagocytosis of IL-4/IL-13-induced M2a microglia was not affected by CB2 KO [166].

CD40 ligands, which are primarily expressed on activated CD4+ T cells, can bind to CD40 receptors on microglia to induce inflammatory responses, such as pro-inflammatory cytokine secretion and cytotoxic radical production. Microglia typically exhibit low CD40 expression in the healthy brain. However, when activated from certain triggers such as brain damage, CD40 expression is increased, which is known to be associated with the development of MS and the promotion of intracerebral inflammation [202,203]. In mouse primary microglia, JWH-015 (≥1.25 μM) reduced CD40 expression induced by IFN-γ, as well as significantly suppressed activation of the JAK/STAT pathway under IFN-γ stimulation [162]. In addition, JWH-015 (5 μM) reversed CD40-mediated inhibition of AD-associated amyloid β (Aβ) peptide phagocytosis over a 3 h incubation, and suppressed the production of inflammatory factors, TNF and NO [162]. However, given the low CB2-selectivity of JWH-015, the concentrations used could have induced activity via targets other than CB2, such as CB1. A number of other AD studies have included assessment of the role of CB2 in Aβ clearance. These are discussed in Section 6.3.

Lenabasum (3 μM), a clinically trialled slightly CB2-selective synthetic agonist (see also Section 7.2, Table 3), exhibited no significant effect on the function of MDMs from healthy individuals. However, it enhanced phagocytosis in primary MDMs (differentiated for GM-CSF for 6 days) from patients with cystic fibrosis (CF) monitored over a 90 min duration [204]. In vitro macrophage models for cirrhosis [205] and atherosclerosis [206] also found that CB2-selective agonists reversed inflammatory stimuli-induced suppression of phagocytosis or efferocytosis (see also Section 6.5).

In contrast to most other reports, 2-AG (10 μM) for 24 h modestly suppressed undifferentiated BV-2 cell phagocytosis of zymosan, which was recovered by ERK1/2 inhibitor U0126 [207]. However, the relatively high ligand concentration used implies that targets other than CB2 could have been involved. Another study using mouse macrophage cell lines (unstimulated J774A.1 and RAW264.7) and mouse peripheral blood-derived macrophages found that CB2 activation by JWH-133 (0.1–10 μM, 1 h) had no significant effect on phagocytic activity of zymosan particles (despite CB2 expression having been confirmed), whereas CB1 activation enhanced phagocytosis through the G(α)i/o–RhoA–ROCK pathway [208]. These discrepancies highlight the complexity of CB2-mediated effects, which may depend on macrophage origin, activation status, nature of the phagocytic stimulus, activating ligand, and other factors.

Cannabinoids have potential to influence not only macrophage phagocytosis but also the antigen presentation pathway. Mouse macrophages (hybridoma line) were incubated with a native antigen (lysozyme) that required processing prior to MHC presentation, versus a pre-processed synthetic antigen, and assessed for the ability to activate T helper cells (indicated by IL-2 secretion) [209]. Macrophages incubated with either antigen stimulated T cell IL-2 secretion. However, when macrophages were pre-treated with non-selective cannabinoid agonists THC (0.1–100 nM) or CP55,940 (0.1 nM–1 μM), only macrophages loaded with the synthetic pre-processed antigen fully activated T cells, whereas the ability of native antigen-loaded macrophages to activate T cells was impaired. These findings indicate that cannabinoids can specifically interfere with antigen processing, without affecting peptide presentation [209]. Furthermore, CB2 but not CB1 mRNA was detected in the macrophages, and the suppressive effect of THC was not reversed by CB1-selective inverse agonist SR141716A (SR1), whereas SR2 completely abrogated the suppression. In the same model, THC enhanced aspartyl cathepsin D activity (without effect on other enzymes in the processing pathway, nor MHC expression), which indicated a change in antigen processing profile, rather than an overall deficiency in processing, which would likely have antigen-specific implications [210]. Indeed, THC had previously been found to inhibit processing of lysozyme at the same time as processing of cytochrome c was enhanced and ovalbumin was unchanged, though the involvement of CB2 was not tested in this study [211]. This suggests that CB2 signalling may regulate adaptive immunity by modulating macrophage antigen-processing [209].

### 5.2. Inflammasome and Autophagy

Both CB2-selective and non-selective cannabinoid agonists have been consistently found to promote autophagy while suppressing the initiation and activation of inflammasome, thereby alleviating inflammation in various inflammatory disease models.

Synthetic CB2-selective agonists, HU308, AM1241, and JWH-133, exhibited similar anti-inflammatory effects by promoting autophagy and reducing inflammasome formation across various types of immune environments. Along with this, the distinct features observed in each immune response further support a reciprocal relationship among CB2 signalling pathways, inflammasome activity, and autophagy.

In both cecal ligation puncture (CLP)-induced septic mice models and LPS/ATP-stimulated murine bone marrow-derived macrophages (BMDMs), the inhibition of pyroptosis via CB2 activation was assessed using HU308- and CB2-selective inverse agonist AM630 [212]. Treatment with HU308 (10 μM) decreased NLRP3 protein levels and reduced activation of caspase-1 and GSDMD, resulting in suppression of pyroptosis. These reductions were abolished by AM630. Similar effects on pyroptosis were observed in murine BMDMs treated with LPS and ATP. In a stable CB2 knockdown cell line derived from murine BMDMs, the HU308-induced reduction in pyroptosis was absent and also unaffected by AM630, further supporting that endogenous CB2 is essential for cannabinoid-mediated cellular protection [212]. In a septic lung injury study with a CLP-induced mouse model, HU-308 (2.5 mg/kg i.p. shortly after CLP induction) upregulated the level of autophagy-related protein, resulting in the downregulation of pro-inflammatory cytokine mRNA levels and attenuation of lung injury [213]. Similarly, treatment with HU308 (10 μM) in RAW264.7 cells increased the LPS-induced expression of autophagy-associated mRNAs and proteins, while suppressing the pro-inflammatory cytokines release and NLRP3 mRNA expression. The effect of HU308 was blocked by the autophagy inhibitor, 3-MA, suggesting that the protective effect of CB2 activation is via enhancing autophagy [213]. However, this concentration of HU308 was relatively high and CB2-dependence was not confirmed by inverse agonist competition.

HU308 (10 μM) was also found to inhibit and enhance autophagy in mouse peritoneal macrophages treated with LPS/DSS [214]. These effects were absent in macrophages isolated from CB2 KO mice, supporting the CB2-mediated mechanism. Notably, siRNA knockdown of autophagy-related gene 5 (Atg5) in peritoneal macrophages significantly attenuated the inhibitory effect of HU308 on LPS/DSS-induced NLRP3 inflammasome activation. In vivo, HU308 (1 mg/kg/day orally [p.o.] in water [aq.], ad libitum [ad lib.] for 8 days) alleviated DSS-induced colitis, which was associated with reduced colonic inflammation and suppression of NLRP3 inflammasome activation in wild-type mice. In contrast, CB2 KO mice exhibited more severe inflammation and greater NLRP3 inflammasome activation following DSS administration compared to wild-type controls. The AMPK-mTOR-P70S6K signalling pathway was involved in the CB2 mediated regulation of autophagy and inflammasome activity [214].

Comparable findings were obtained in the EAE MS model through both CB2 KO mice and HU308 treatment (1 mg/kg/day i.p. from day 3 after EAE induction) [215]. Additionally, when autophagy was inhibited in BV-2 microglial cells via ATG5-specific siRNA, the anti-inflammatory effect of HU308 (10 µM) was lost, indicating that CB2-mediated anti-inflammatory responses rely on autophagy signalling [215].

In a pilocarpine-induced chronic epilepsy mouse model, treatment with the CB2-selective agonist, AM1241 (1 mg/kg/day i.p. from 8th day after induction of status epilepticus), reduced NLRP3 inflammasome activation and suppressed inflammatory markers in hippocampal microglia, which was accompanied by decreased neuronal loss, seizure frequency, and depressive-like behaviour in mouse model [216]. These effects were mediated via the AMPK signalling pathway. The role of microglial CB2 in epilepsy is discussed further in Section 6.3.

Chronic alcohol exposure in late adolescent mice induced microglial activation and increased microglia–neuron interactions, accompanied by elevated expression of inflammasome-related molecules including NLRP3, ASC, and caspase-1 [217]. This led to increased secretion of IL-1β, contributing to anxiety-like behaviours. AM1241 (3 or 6 mg/kg i.p. 1 h before alcohol treatment) suppressed NLRP3 inflammasome activation and prevented morphological changes in microglia. In LPS-activated N9 microglia cells, AM1241 (5 μM) inhibited the expression of NLRP3, IL-1β, which was abolished by AM630 [217]. Corresponding results using ATG5-KO mice were demonstrated in an alcohol-induced inflammation and steatosis model, further supporting the protective role of CB2 activation through autophagy induction. JWH-133 (5 μM) activated autophagy via a heme oxygenase-1-dependent pathway and effectively suppressed LPS-induced pro-inflammatory gene expression in mouse Kupffer cells, the resident macrophages of the liver. However, this anti-inflammatory effect was abolished in ATG5-deficient macrophages, indicating that the anti-inflammatory action of CB2 receptors relies on autophagy. However, as JWH-133 was used at a relatively high concentration and the CB2-dependence of JWH-133 effects were not supported by inverse agonist treatment, the involvement of alternative targets cannot be excluded [218].

Similar effects were reported for phytocannabinoids THC (5 μM) and CBD (5 μM), which suppressed the expression of NLRP3 inflammasome-associated proteins (NLRP3, pro-caspase-1, and pro-IL-1β) as well as the secretion of mature IL-1β in THP-1 macrophages stimulated with LPS and ATP [219]. Different pathways of regulation were indicated, with CBD inhibiting phosphorylation of the NF-κB p65 subunit at Ser-536, whereas THC suppressed NLRP3 inflammasome activation via an NF-κB-independent mechanism [219]. However, whether the mechanisms of action for THC and/or CBD involved CB2 were not investigated, and multiple alternative targets and/or indirect action via modulation of endocannabinoid enzymes are also possible [104,109].

### 5.3. Migration

Activation of CB2 can modulate migration and infiltration of macrophages into inflammatory regions, including infection sites.

The accumulation of 2-AG is endogenously regulated by the degrading enzyme, ABHD6. In BV-2 microglia, shRNA knockdown of the ABHD6 gene reduced 2-AG hydrolysis and enhanced 2-AG-induced cell migration (EC_50_ ~120 nM) [220]. This modulatory effect of 2-AG was mediated by CB2 activation, as verified by pre-treatment with SR2. 2-AG can facilitate cell migration by promoting adhesion in several types of macrophages, including HL-60, U937, and BV-2 cell lines, mouse primary microglia, and human primary monocytes [221,222,223]. In addition to regulating the migration of macrophages themselves, 2-AG also enhances HL-60 cell secretion of chemoattractants, including IL-8 and monocyte chemoattractant protein-1 (MCP-1) [224]. These effects were observed to be dependent on CB2 as they were blocked by pre-treatment with SR2 [223,224].

In contrast, pre-treatment with THC either in vivo (25 or 50 mg/kg i.p.) or in vitro (1 pM–1 μM) significantly inhibited the RANTES/CCL5-induced chemotactic response of murine peritoneal macrophages [225]. Similar in vitro results were observed for synthetic non-selective agonist CP55,940 (10 pM–1 μM) and CB2-selective agonist O-2137 (100 pM and 10 nM–10 μM). As well as the ligands acting with surprisingly high potency, responses did not reflect a typical concentration–response relationship, perhaps indicating the involvement of multiple targets and/or feedback/-forward mechanisms. Nonetheless, the inhibitory effect of CP55,940 on chemotaxis was indicated to be largely CB2-dependent, as it was reversed at moderate concentrations (10 pM–10 nM) by SR2, but not SR1. Furthermore, the inhibitory effect of THC on chemotaxis was absent in macrophages from CB2 KO mice, though RANTES-induced migration was blunted in comparison with wild-type mice [225].

CP55,940 (10 nM–1 μM) suppressed both spontaneous migration and formyl-methionyl-leucine-phenylalanine (fMLP)-induced chemotaxis in rat peritoneal macrophages [226]. These effects were concentration-dependent and were inhibited by a CB2 antagonist, while a CB1 antagonist only inhibited spontaneous migration. In a comparable manner, CBD (10 nM–10 μM) reduced fMLP-induced chemotaxis of murine macrophages, which was prevented by a CB2 antagonist [227]. JWH-015 (5–20 μM) and JWH-133 (10 μM), slightly and moderately CB2-selective agonists, respectively, attenuated CCL2 and CCL3-induced migration of human primary monocytes along with reducing the expression of chemokine receptors CCR2 and CCR1 [228]. These agonists also inhibited expression of adhesion molecule ICAM-1 on IFN-γ-induced human monocytes. Although the JWH-015 concentrations used were unlikely to have been selective for CB2, effects were blocked by co-incubation with SR2 but not SR1. These modulatory effects of JWH-015 were found to depend on PI3K/Akt and ERK1/2 signalling pathways [228].

In the early stages of HIV infection, infected cells secrete trans-activating protein (Tat), which acts as a chemoattractant in inflammatory responses and promotes the infiltration of various immune cells, including macrophages. CB2 agonists have been reported to inhibit Tat-induced migration toward HIV-1-infected cells. In U937 cells, both THC (EC_50_ ~100 nM) and CP55,940 (EC_50_ ~30 nM) significantly attenuated cell migration toward Tat in a concentration-dependent manner [229]. The inhibitory effect of CP55,940 was reversed by SR2 (concentration–response curve rightward shift), whereas treatment with SR1 had no effect. Further supporting a central role of CB2 in the response, CB2-selective agonist O-2137 also suppressed Tat-induced migration (EC_50_ ~50 nM), and CB2 siRNA knockdown abolished the inhibitory effect of THC [229]. Similar findings were observed in BV-2 cells, where THC (100 nM and 10 μM), CP55,940 (1 nM–10 μM), and 2-AG (1 and 10 μM) suppressed Tat-induced migration in a concentration-dependent manner; all effects on migration were blocked by SR2 but not SR1 [230].

Atherosclerosis is a chronic inflammatory disease characterised by the accumulation of lipids, immune cells, and connective tissue within the arterial wall. In the early stages, monocytes adhere to injured endothelium through adhesion molecules such as P-selectin, VCAM-1, and ICAM-1. After transmigration, monocytes differentiate into foam cells, contributing to plaque formation. The effect of non-selective agonist WIN55,212-2 on atherosclerosis progression was investigated in ApoE-KO (ApoE^−/−^) mice [231]. Treatment with WIN55,212-2 (0.5 or 1 mg/day i.p. from 12 weeks of age) reduced macrophage infiltration and the expression of P-selectin, VCAM-1, and ICAM-1 in plaque lesions, leading to smaller atherosclerotic lesions. Consistent results were observed in in vitro experiments using HUVEC and U937 cells, and CB2-dependent effects were supported due to reversal by AM630 in both the in vivo and in vitro models [231]. THC (1 mg/kg/day p.o. in milk ad lib. for 2–6 weeks) significantly attenuated atherosclerotic plaque formation in ApoE^−/−^ mice, this effect was reversed by SR2 [232]. Furthermore, THC (1.9 nM) suppressed MCP-1 induced migration of peritoneal macrophages isolated from ApoE^−/−^ mice. This suppressive effect of THC was blocked by SR2 and was absent in peritoneal macrophages derived from CB2 KO mice, supporting a CB2-dependent mechanism. Relating to the treatment of atherosclerosis, balloon catheter inflation to restore narrowed arteries can cause vascular injury and restenosis, involving inflammatory cytokine release and immune cell recruitment. In an analogous mouse model, JWH-133 (5 mg/kg/day i.p. starting shortly before injury) inhibited macrophage migration and reduced inflammation [233].

The effect of CB2 activation on macrophage/microglial infiltration in traumatic brain injury (TBI) [234] and Alzheimer’s disease (AD) [235] models will be detailed in Section 6.2 and Section 6.3, respectively.

### 5.4. Cytokine Secretion

The effects of CB2 activation on cytokine production and secretion have been studied in various types of macrophages.

LPS, with or without IFN-γ co-stimulation, has been utilised widely to stimulate pro-inflammatory cytokine release from macrophages and demonstrate inhibition by cannabinoids, in some cases providing evidence for CB2 as the primary mediator. Effective CB2-selective agonists included JWH-133 applied to human (3 μM or 10 μM) [236,237] and mouse (10 nM–5 μM) [238] primary macrophages, AM1241 (2 or 5 μM) on isolated mouse Kupffer cells [239], and (E)-β-Caryophyllene (BCP, 500 nM, a plant-derived cannabinoid) on human primary immune cells in whole blood [121]. Most of these studies supported a CB2-dependent mechanism via CB2-selective inverse agonist sensitivity. In the BCP study, anti-inflammatory effects were absent in CB2 KO mice [121]. As well as suppression of pro-inflammatory cytokines, JWH-133 (10–100 nM) and AEA (5–15 μM) also CB2-dependently promoted the secretion of the anti-inflammatory cytokine IL-10 in LPS/IFN-γ stimulated murine microglia and macrophages [240,241]. IL-10 production and suppression of IL-12 and IL-23 were mediated through the MAPK (ERK1/2 and c-Jun N-terminal kinase, JNK) signalling pathway, while regulation of IL-10 also involved NF-κB [240,241]. AEA (1 μM) reduced NO release in rat microglia, which was partially blocked by a CB2 inverse agonist, but not CB1, GPR18, or GPR55 inverse agonists [165]. 3,4-Methylenedioxymethamphetamine (MDMA, “ecstasy”) can produce neurotoxicity in rats, which is associated with neuroinflammation; in particular, IL-1β production and activation of microglia. JWH-015 (2.4 mg/kg i.p., three doses prior to MDMA) markedly reduced MDMA-induced elevated IL-1β [242]. Expression of a microglial activation marker was also suppressed, which was reversed by AM630.

Non-CB2-selective cannabinoids 2-AG, WIN55,212-2, THC, and CBD have produced similar anti-inflammatory effects, inhibiting the secretion of pro-inflammatory cytokines in human primary MDMs [236,243] or THP-1 macrophages [219]; however, these studies did not investigate whether CB2 was involved in the mechanism of action.

In contrasting findings, in murine peritoneal macrophages, CBD (5–500 nM, but not 1–5 μM) inhibited the release of LPS-induced IL-10 while enhancing the production of LPS + IFN-γ-induced IL-12 through a mechanism involving both CB2 and CB1 [227]. The opposing effects in comparison with the majority of other studies with cannabinoid agonists may relate to the ability of CBD to negatively modulate CB1/CB2 (though effects were partially reversed with CB1/CB2 inverse agonists), the specific context of the model used in this study, or involve CBD’s activity at a wide variety of targets other than CB1/CB2.

Indeed, some evidence indicates that the anti-inflammatory effects of cannabinoids in LPS-stimulated models may be mediated through pathways other than CB1 and CB2. For example, in rat primary microglia LPS-induced gene expression of pro-inflammatory cytokines was inhibited by not only THC (1 and/or 10 μM), CP55,940 (10 μM), and levonantradol (10 μM) but also their respective enantiomers with low affinity for CB1 and CB2 [244]. Moreover, the inhibitory effect of levonantradol was not reversed by either SR1 or SR2. Interpretation of effects from exogenously applied endocannabinoids can also be complex due to the potential involvement of metabolites in inflammatory responses. In J774, macrophages THC (1–10 μM) and AEA (10–30 μM) attenuated LPS-induced IL-6 and NO production, whereas 2-AG (3–30 μM) also reduced IL-6 but slightly enhanced iNOS-dependent NO release [245]. This difference was thought to be due to 2-AG serving as a substrate for cyclooxygenase (COX)-driven prostaglandin E2 (PGE_2_) biosynthesis which, by itself, produced effects analogous to those of 2-AG alone and can have immunomodulatory effects via the PGE_2_ receptor 2 (EP2) [245].

A range of disease models involving inflammatory mechanisms have also been investigated.

In a mouse model of stroke, activation of CB2 by JWH-133 (1.5 mg/kg i.p. shortly after occlusion) reduced the extent of brain damage and improved motor function [188]. These effects were shown to be CB2-dependent by using a CB2 antagonist and CB2 KO mice. JWH-133 also decreased the number of activated microglia and/or infiltrated macrophages in injured cerebral cortex, as well as reduced gene expression of both pro-inflammatory cytokines (IL-6, TNFα, MCP1α, and iNOS) and certain anti-inflammatory cytokines (IL-10, TGFβ, and Ym1) [188]. In a mouse model of hepatic ischemia–reperfusion injury, levels of AEA and 2-AG were elevated in hepatocytes, Kupffer cells, and endothelial cells [246]. The upregulated levels were associated with increased tissue damage and higher concentrations of pro-inflammatory cytokines, including TNF, MIP-1α, and MIP-2. JWH-133 (20 mg/kg i.p., 1 h prior to occlusion injury) reduced immune cell infiltration, pro-inflammatory markers and the expression of adhesion molecules in serum and liver, and the reversal of these effects by SR2 supported that the responses were CB2-dependent [246]. Complementary evidence showed that BCP (5 μM) suppressed pro-inflammatory cytokine production in hypoxia-exposed BV-2 cells. This inhibitory effect of BCP was abolished by CB2 siRNA knockdown [247]. These findings suggest that CB2 activation may help suppress excessive immune responses following stroke and other ischaemia-related conditions.

Mycobacterium tuberculosis (MTB) is a serious infectious disease that is difficult to treat due to immune evasion and antibiotic resistance. Mycobacteria regulate the response and metabolism of macrophages after infection, allowing them to survive and proliferate inside the cells. In J774 cells infected with irradiated *Mycobacterium bovis*-BCG (iBCG), pre-treatment with three CB2 selective agonists (GP1a, JWH-133, and GW833972A) at 10 μM reduced the secretion of TNF and IL-6 induced by iBCG [248]. GP1a pre-treatment also inhibited the production of inflammatory mediators, such as PGE_2_ and NO, and reduced the transcription of inflammatory-related genes (iNOS, IL-1β, COX-2) as well as lipid metabolism-related genes. Moreover, GP1a pre-treatment inhibited activation of the NF-κB signalling pathway. GP1a effects were at least partially blocked by AM630, indicating CB2 involvement in the mechanism of action [248].

In the spinal cords of MS model TMEV-infected mice, AEA (3.5 mg/kg over 7 days via subcutaneous pump, after onset of symptoms) suppressed IL-12p70 and IL-23 mRNA expression, as well as enhanced circulating IL-10. AEA treatment also produced an improvement in motor disturbances [249]. In isolated microglia infected by TMEV, AEA (10 μM) had similar effects that were partially blocked by a CB2 inverse agonist [249].

Apoptotic cancer-conditioned medium induced a TAM-like (tumour-promoting) phenotype in human MDMs, which exhibited increased secretion of IL-10, IL-6, and IL-8, alongside suppressed IL-12 production [250]. RNAi screening demonstrated CB2 as a key regulator in TAM differentiation. Notably, treatment with 10 µM AM630 or CB2 siRNA knockdown significantly reduced IL-10 and IL-6 production. These findings indicate that CB2 activation may contribute to TAM differentiation and generating tumour-promoting immune environments, implicating CB2 inverse agonism as a potential therapeutic strategy in cancer. The role of CB2 in TAMs is discussed further in Section 6.6.

### 5.5. Differentiation and Polarisation

CB2 activity can influence the differentiation of monocytes into macrophages and their subsequent M1/M2 polarisation. This extends to the regulation of macrophage reprogramming under pathological conditions, such as differentiation to TAMs and foam cells. In addition, polarisation-inducing stimuli reciprocally regulate endocannabinoid synthesis, which can establish a feedback loop.

In cell-based in vitro models, JWH-133 (5 μM pre-incubation) has been found to inhibit M1 and promote M2 macrophage polarisation, including in LPS-stimulated RAW264.7 cells [251], zymosan-stimulated rat primary Kupffer cells [252], IL-17 induced RAW264.7 cells [253], and LPS stimulated thioglycollate-induced murine peritoneal macrophages [254], though none of these studies tested specifically for CB2-dependence of the effects. However, consistent with these findings, CB2 KO from LPS-stimulated mouse primary Kupffer cells enhanced expression of some M1-related genes, and reduced expression of some M2 genes [251].

Similar findings have been demonstrated in injured tissue models. In a study investigating the infiltration of M1 and M2 macrophages into incision wounds on mouse skin, JWH-133 (3 mg/kg/day i.p. from incision) produced a significant reduction in M1 macrophage infiltration, M1 marker expression, and pro-inflammatory cytokine production rather than promoting M2 macrophage polarisation [255]. JWH-133 also induced changes consistent with an M1 to M2 phenotype shift in rodent models for acute (20 mg/kg [i.p.?], two doses prior to liver injury [254]) and chronic (10 mg/kg single dose i.p. between liver injury and LPS-induced Kupffer cell activation [252]) liver disease. Beneficial effects on alcoholic liver disease and hepatocyte steatosis induced by an alcohol diet in mice were also produced by JWH-133 (3 mg/kg/day i.p., with alcohol feeding), and this coincided with the inhibition of M1 polarisation of Kupffer cells, though expression of some M2 markers were also blunted [251]. CB2 KO mice exhibited enhanced M1 marker gene levels and reduced M2 markers. The functional importance of CB2 was further demonstrated in a skeletal muscle ischaemia–reperfusion injury mouse model utilising CB2 KO [256]. CB2 KO mice exhibited enhanced infiltration of M1 macrophages and elevated expression of associated proteins, while presence of M2 macrophages was reduced, correlating with significantly impaired regeneration of damaged muscle fibres. Moreover, in vitro co-culture experiments demonstrated that CB2 KO macrophages exhibited enhanced M1 polarisation and reduced M2 polarisation, which in turn hindered myoblast differentiation [256].

Macrophage polarisation in patients with chronic inflammatory diseases can also be modulated by CB2 activation. Celiac disease has been suggested to involve macrophages in the pathophysiology, along with chronic inflammation in intestinal epithelia. MDM from celiac disease patients were characterised as more pro-inflammatory based on the evaluation of macrophage markers and profile of cytokine production, and expressed less CB2 compared to healthy donors [257]. JWH-133 (100 nM)-induced CB2 activation converted the macrophage phenotype of celiac disease patients from M1 to M2 type, which was associated with a decrease in intestinal damage. In MDMs from paediatric patients with IBD, CB2 expression was reduced, and the balance between M1 and M2 macrophages was skewed toward the pro-inflammatory M1 phenotype, indicating a hyperinflammatory state [198]. Stimulation with JWH-133 (100 nM) promoted M2 polarisation and improved intestinal barrier function. A study on human lung-resident macrophages isolated from macroscopically normal areas of resected lung tissue from lung adenocarcinoma patients demonstrated that LPS stimulation increased the production of 2-AG [258]. JWH-133 (1 μM) led to ERK1/2 phosphorylation and generation of ROS while suppressing the LPS-induced secretion of angiogenic and lymphangiogenic factors. These inhibitory effects were abolished by AM630 treatment. These findings imply potential involvement of CB2 in modulating M2-like macrophage functions related to vascular remodelling.

In neuroinflammation, CB2 activation has again been reported to shift microglial polarisation away from M1 and toward M2 phenotypes. In LPS and IFN-γ-induced pro-inflammatory N9 microglia, treatment with AM1241 (10 μM) downregulated M1 markers such as iNOS and TNF while upregulating the expression of Arg-1, an M2 marker, though it was not verified in this study that CB2 was the primary contributor to these effects [259]. In the APP/PS1 (chimeric mouse/human APP with mutant human presenilin 1) AD transgenic mouse model, chronic administration of JWH-015 (0.5 mg/kg i.p., daily for 8 weeks) reduced Iba1 expression in the cortex and promoted a shift in microglial phenotype from M1 to M2 type [260]. Whether the effects from this only ~28-fold CB2-selective agonist were CB2-mediated was not tested. Further findings relating to CB2 modulation of macrophages/microglia in AD, and related symptoms and pathology, are discussed in Section 6.3.

Similar effects of CB2-selective agonists on microglial polarisation were reported from two MS models. In microglia isolated from an EAE mouse model treated with BCP (5 mg/kg p.o. daily from 10 days after EAE induction), MS-associated inflammatory markers were shifted toward an anti-inflammatory profile [261]. This reflected a transition from M1 toward M2 polarisation, which was accompanied by conversion of T lymphocytes from pro- to anti-inflammatory phenotypes. CB2-dependence was not specifically tested in this study. BCP treatment also alleviated general clinical symptoms in EAE mice (see also Section 6.3). In TMEV-induced neuroinflammation, 2-AG (5 mg/kg/day i.p. for 7 days from infection) attenuated microglial activation and promoted their shift toward an anti-inflammatory phenotype [262]. CB2-dependence of the response was supported through comparison of AM630 and AM251 (CB1-selective inverse agonist) administration.

Neurological damage in traumatic brain injury (TBI) is also exacerbated by inflammation mediated by microglia. Interestingly, both CB2 agonists [234,263] and an inverse agonist [264] have shown potential for producing therapeutic effects by shifting microglia from M1-like to M2-like phenotypes. These findings are detailed in Section 6.2.

Beyond modulating the balance between M1 and M2 polarisation, M2 subtype polarisation is also influenced by CB2 activation and involves regulation of the endocannabinoid system. IL-4/IL-13-induction of rat primary M2a microglia enhanced 2-AG concentration, which correlated with enhanced expression of a 2-AG synthesising enzyme and reduced expression of a degrading enzyme [166]. After 6 h of IL-4/IL-13 stimulation, CB2 expression was increased. By 24 h, CB2 expression remained elevated, accompanied by an upregulation of CB1. Treatment of undifferentiated rat or human primary microglia with 1 nM (but not 100 nM) 2-AG or AEA significantly increased the protein level of Arg1, as well as gene expression of Arg1 and suppressor of cytokine signalling 3 (SOCS3). CB1 and CB2 antagonists both blocked Arg1 expression during M2a polarisation, suggesting that signalling through both CB receptors was important for M2a formation. Microglia from CB2 KO mice also had morphological differences from wild-type cells and expressed significantly less Arg1 both basally and after IL4/IL-13 stimulation, further supporting an important role for CB2 in microglial differentiation and polarisation. TGF-β–induced M2c macrophages exhibited enhanced AEA levels and, again, gene expression for AEA synthesising and degrading enzymes were modified. After a 6 h stimulation with TGF-β, the expression of both CB1 and CB2 was markedly increased, but both were reduced again after 24 h [166].

Pathological environments, such as the tumour microenvironment and hyperlipidemic conditions, can promote the formation of specialised macrophages (e.g., TAMs and foam cells), whose function is also influenced by CB2 activation. In TAMs isolated from a tumour cell-injected mouse model, monoacylglycerol lipase (MAGL) KO (and presumably reduced degradation of 2-AG) led to enhanced CB2/TLR4-dependent acquisition of an M2-like phenotype, promoting tumour progression [265]. Treatment with AM630 (0.3 mg/kg/day i.p. for 8 weeks) delayed tumour growth in both transplanted and genetic cancer models. Moreover, reduced MAGL expression in human tumours correlated with reduced survival in patients. These findings suggest that MAGL acts as a negative regulator of CB2-mediated pro-tumoural functions of TAMs, and indicate that blocking CB2 to may be beneficial in this context [265]. Consistent with these findings, in mice with transplanted GL261 cell glioblastoma, AM630 administration (5 mg/kg i.p. every second day from 1 week after cell implantation) resulted in a reduction in TAMs and microglia within the tumour, whereas GW405833 (5 mg/kg i.p., same dosing schedule) did not affect the population of TAMs. In addition, AM630-treated glioblastoma mice showed an improvement in survival rate (see also Section 6.6) [266].

The transition of macrophages into foam cells is mediated by uncontrolled uptake of oxLDL. Activation of CB2 in PMA-differentiated THP-1 cells by AEA (2.5 μM), 2-AG (5 μM), or JWH-015 (50 nM) reduced oxLDL accumulation, whereas CB1-selective agonist ACEA had no effect [267]. JWH-015 also suppressed inflammatory cytokine production, which was sensitive to blockade by SR2 but not SR1, consistent with CB2 agonists having potential for lowering atherosclerosis risk.

## 6. Therapeutic Effects Mediated by CB2 Activation on Macrophages

Although the effects of CB2 activity on the typical functions of macrophages and its regulation of specific signalling pathways remain incompletely understood, several studies have demonstrated therapeutic effects of CB2-targeted compounds mediated through macrophages, particularly microglia, in the contexts of infectious diseases, neuropathic pain, neurodegenerative diseases, and autoimmunity, as well as non-neuronal organ injury, chronic inflammation, and cancer.

### 6.1. Infectious Diseases

Multiple studies have investigated the role of CB2 in macrophages and microglia under HIV-infected conditions. THC (1–30 μM) applied during the differentiation of human primary monocytes into macrophages prevented subsequent HIV-1 infection in a concentration-dependent manner [268]. This inhibitory effect on HIV-1 infection was not observed when THC was applied to already-differentiated macrophages. THC (30 μM) acted on the early stages of infection by downregulating the expression of key receptors required for viral entry (CD4, CCR5, and CXCR4). Furthermore, THC promoted a more infection-resistant state by modulating macrophage phenotype and the expression of viral inhibitory factors. The suppressive effect of THC on HIV-1 infection was reproduced to a lesser extent by CP55,940 (10 μM) and JWH-133 (1 and 10 μM), but not ACEA (CB1-selective agonist) or O-1602 (GPR55-selective agonist) [268]. Combined, these findings may support a CB2-involving mechanism, though the low potency and efficacy of CP55,940 and JWH-133 are surprising. The antiviral effect of CB2 activation on HIV infection has also been demonstrated in human microglial cells isolated from foetal brain tissue, where treatment with WIN55,212-2 and JWH-015 at 1 μM suppressed HIV replication [269]. These effects were reversed by SR2, but not CB1-selective inverse agonists AM251 or SR1. Given that HIV primarily enters microglia via CCR5, the downregulation of CCR5 expression following WIN55,212-2 treatment further supported the antiviral effect by limiting viral entry.

HIV-associated neurocognitive disorders (HAND) are linked to synaptic damage and neuron loss, which gradually reduce learning and memory abilities, ultimately leading to cognitive dysfunction. In brain tissue from patients with HIV encephalitis (HIVE) and HIV-associated neurological comorbidities, the expression of both CB1 and CB2 was increased in perivascular macrophages and microglia [270]. This indicates that CB2 is available to potentially serve as a therapeutic target for neuroinflammation and neurotoxicity. Although there is still no specific treatment for HAND, the HIV-1 envelope glycoprotein gp120 has been identified as a major pathogenic factor contributing to neuronal injury and development of HAND. Among other changes, gp120 activates microglia. In gp120-injected rats, inflammatory changes in the hippocampus included significantly increased mRNA expression of pro-inflammatory cytokines (IL-1β, IL-6, TNF, and CXCL10), enhanced IL-1β protein and reduced anti-inflammatory cytokine IL-10 protein [271]. gp120 also upregulated pro-apoptotic gene expression and downregulated anti-apoptotic factors, likely via the p38/JNK MAPK signalling pathway. However, pre-treatment with WIN55,212-2 (3 mg/kg/day [i.p.?] for 3 days) significantly reduced gp120-induced spatial learning and memory deficits, as well as the expression of inflammatory, apoptotic, and p38/JNK genes in the hippocampus. These effects were reversed by co-administration of AM630, supporting that the action of WIN55,212-2 is mediated through CB2 activation [271]. Furthermore, another study reported that gp120-induced neurotoxicity particularly affects human dopaminergic neurons, resulting in decreased dopamine uptake, increased neuronal death, and elevated lipid peroxidation. Studying human primary neural/glial co-cultures (from foetal tissue), treatment with WIN55,212-2 (0.3 μM) alleviated these toxic effects via a CB2-dependent mechanism, as evidenced by SR2 blockade and replicated by JWH-015 (1 μM) [272]. The migration of primary human microglia was suppressed by WIN55,212-2 (1 μM) through a CB2-mediated pathway (reversed by SR2 but not SR1).

HIV-infected human primary MDMs secrete excessive levels of cathepsin B (CATB), which contributes to neuronal toxicity and the pathophysiology of HAND. JWH-133 (0.5 μM) suppressed both HIV-1 replication and CATB secretion in MDMs monitored 3 to 12 days post-infection, as well as reduced neuronal apoptosis (neuroblastoma co-culture) [273]. The first two effects were blocked by SR2 (apoptosis not tested). HU308 (5 μM for HIV replication; 10 μM for CATB secretion) produced similar outcome as JWH-133, but to very small extents and only at one acute timepoint. The mechanism of CATB neurotoxicity involves NF-κB activation, oxidative stress, and lysosomal exocytosis. Again in HIV-1-infected human primary MDMs, JWH-133 (0.5 μM) downregulated the expression of CATB, NF-κB, Nrf2-mediated oxidative stress response proteins, and lysosome pathway-related proteins that were otherwise upregulated by HIV infection, though CB2-dependence of these effects was not verified [274]. Collectively, these findings suggest that CB2 agonists may be protective in HIV infection, both for preventing infection and exerting neuroprotective effects in conditions such as HAND.

In MTB, upon mycobacterium infection, host macrophages shift their energy metabolism from glucose to fatty acids, a process accompanied by the formation of lipid droplets. These lipid droplets not only further promote inflammation but also contribute to the formation of foamy macrophages, which provide a niche where MTB can evade immune responses and proliferate. Therefore, MTB treatment efficacy may be enhanced via regulating the expression of lipid metabolism-related genes and reducing lipid droplet accumulation. In J774A.1 macrophages, GP1a, JWH-133, and GW833972A at 10 μM all demonstrated inhibitory effects on the secretion of pro-inflammatory cytokines [248]. Only GP1a was further investigated for its effects on lipid metabolism, which suppressed the transcription of lipid metabolism-related genes and inhibited lipid droplet formation. These effects of GP1a were reversed by AM630 and were associated with modulation of the NF-κB signalling pathway.

Cerebral malaria, an often-fatal complication caused by *Plasmodium falciparum* infection, involves parasite sequestration, disruption of the blood–brain barrier, and intense inflammation in the brain. Contrasting with findings from other infectious disease models, in mice infected with malaria, CB2 KO resulted in increased survival rates and reduced blood–brain barrier damage [275]. In CB2 KO, macrophages in the spleen exhibited enhanced anti-inflammatory responses and decreased expression of pro-inflammatory cytokines. Expression of CCL17, an M2 macrophage-derived chemokine, was essential for improved survival in CB2 KO mice. Furthermore, SR2 (25 μg/day i.p.) administered to wild-type mice enhanced survival following malaria infection to a similar degree as CB2 KO. While the specific mechanism for these seemingly surprising results is unknown (though it might involve adaptive changes due to chronic KO, as has been hypothesised to be a factor for CB2 KO in AD models; see Section 6.3), these findings indicate the potential for context-dependent outcomes from CB2 modulation.

### 6.2. Neuropathic Pain and Nerve Injury

Nerve injury induced by infection or physical damage, such as traumatic brain injury (TBI) or surgical intervention, is frequently accompanied by neuroinflammation and neuropathic pain. These responses are associated with the activation of resident microglia and infiltration of peripheral macrophages. Furthermore, the activation of microglia and macrophages exacerbates secondary neuronal damage through the release of neurotoxic mediators within both the central and peripheral nervous system. Nerve injury-induced neuropathic pain is often difficult to manage, even with potent analgesics.

LPS/IFN-γ stimulation of THP-1 cells increased neurotoxic secretions that compromised the viability of SH-SY5Y, a human neuroblastoma cell line [276]. JWH-015 (5 μM) elicited anti-inflammatory responses in LPS/IFN-γ-stimulated THP-1 cells and human microglia, reducing neurotoxicity. This effect of JWH-015 was prevented by SR2, but not SR1. THC (5 μM) produced similar protection from neurotoxicity as JWH-015. In contrast, AEA (1, 5, and 10 μM) had no significant effect. Importantly, JWH-015 (1–10 μM) did not alter viability when added directly to SH-SY5Y cells, whereas THC (5 μM) induced neurotoxicity via CB1 (reversed by SR1, but not SR2) and AEA (50 μM) also produced toxicity, which was enhanced by an inhibitor of enzymatic hydrolysis [276]. A subsequent study compared the neurotoxic effects of conditioned media from LPS/IFN-γ-stimulated SIM-A9 cells treated with selective CB1 or CB2 agonists and a non-selective agonist. Inflammatory cytokine mRNA expression and NO induced by LPS/IFN-γ were suppressed by ACEA (1.2 μM or EC_50_ ~ 680 nM, respectively) and HU308 (2.5 μM or EC_50_ ~690 nM). CP55,940 (1.8 μM or EC_50_ ~560 nM) had lesser efficacy for reducing NO and inflammatory cytokine mRNA than the selective agonists, with combined ACEA and HU308 treatment also having lesser effects than these agonists applied alone. Conditioned media from LPS/IFN-γ-induced microglia induced neuronal cell (ST*Hdh*^Q7/Q7^) death, which was prevented by treatment of microglia with all three CB2 agonists. However, receptor subtype-dependence of these effects were unclear, with CB1-versus CB2-selective inverse agonists having some unexpected interactions with agonist responses. This study indicated that activation of CB1 or CB2 receptors separately, rather than simultaneously, may be effective in modulating microglial inflammation and providing neuroprotection [171].

iNOS and ROS are commonly measured as inflammatory markers but are fundamentally cytotoxic factors, distinct from cytokines. In microglia, the resident macrophages of the CNS, iNOS, and ROS induction may regulate cytotoxicity. In LPS-induced BV-2 microglia, iNOS induction was reduced by cannabinoids including AEA and ACPA (a CB1 selective agonist) at 100 nM and 1 μM, as well as AM1241 at 10 nM, 100 nM, and 1 μM [277]. However, the attenuated iNOS induction was not reversed by their respective antagonists. In addition to iNOS induction, ROS generation was suppressed by not only AEA, ACPA, and AM1241 at 100 nM but also SR1 and SR2. Since both CB2-selective agonists and inverse agonists as well as CB1- or non-selective agonists induced apparently similar anti-inflammatory effects in these LPS-stimulated BV-2 microglia, more precise mechanistic studies and comparative research are required to better elucidate the role of CB2 signalling in this model.

In mice with sciatic nerve injury, transgenic overexpression of CB2 suppressed both neuropathic pain and microglial activation [278]. In contrast, wild-type mice that received bone marrow transplants from CB2 KO mice exhibited exacerbated pain responses. These findings suggest that CB2 expressed in bone marrow-derived cells contributed to the development of neuropathic pain in the spinal cord, indicating that CB2 modulates pain through hematopoietic cell-mediated immune mechanisms. Mirror-image pain refers to a phenomenon where neuropathic pain can spread from the site of the injury to the contralateral side. Mice with partial sciatic nerve injury did not spontaneously develop mirror-image allodynia [279]. However, both systemic and myeloid cell-specific CB2 KO mice did develop mirror-image allodynia, whereas this phenotype was absent in mice lacking neuronal CB2 expression. These findings, along with exacerbated spinal cord microgliosis in CB2 KO mice, indicated that CB2-mediated pain modulation occurs via microglial rather than neuronal cells. Relatedly, CB2 KO mice exhibited IFN-γ expression beyond the injury site, worsening the pain response and contributing to spread [280]. Mice deficient in both CB2 and IFN-γ did not exhibit the heightened pain hypersensitivity observed in CB2 KO mice. Additionally, in BV-2 microglial cells, IFN-γ-induced iNOS and CCR2 gene expression was modulated by CB2 receptor activation.

In a mouse spared nerve injury model, repeated administration of CB2 agonist NESS400 (4 mg/kg/day i.p., from injury) reduced both mechanical and thermal hyperalgesia (dose-dependently and blocked by AM630 but not CB1-selective inverse agonist AM251), suppressed the pro-inflammatory activation of spinal microglia, and increased the expression of anti-inflammatory genes, thereby contributing to the alleviation of neuropathic pain [281]. In another neuropathic pain rat model, induced by spinal nerve transection, CB2 was primarily expressed on microglia and perivascular cells, and JWH-015 (two 10 μg/injections intrathecally, four days after transection) attenuated pain hypersensitivity [282]. This analgesic effect was blocked by AM630 but not by CB1-selective inverse agonist AM281, and JWH-015 did not induce behavioural side effects or analgesic tolerance, suggesting that spinal CB2 activation may represent a safe and effective therapeutic strategy for chronic pain. Furthermore, in a rat model of central sensitisation induced by the chemotherapeutic agent paclitaxel, CB2 agonist MDA7 (15 mg/kg/day i.p. for 14 days, starting with paclitaxel) mitigated pain behaviour, suppressed microglial activation, reduced the production of pro-inflammatory mediators, and downregulated the overexpression of brain-derived neurotrophic factor (BDNF) [283]. MDA7 also upregulated the expression of IL-10 and inhibited the expression of genes associated with pain hypersensitivity, further supporting the potential of CB2 activation as a promising approach for the management of chronic pain.

Trifluoro-icaritin (ICTF) is a derivative of an active component from the plant *Epimedium* that is used in traditional Chinese medicine for anti-inflammatory properties. ICTF (3 mg/kg/day i.p.) significantly alleviated pain-related behaviours after 14–21 days of treatment and exhibited anti-inflammatory effects in the spinal cord in a complete Freund’s adjuvant (CFA)-induced chronic inflammatory pain rat model. ICTF increased the expression of CB2, IL-10, and β-endorphin in microglia, while reducing the co-localisation of the microglial marker Iba-1 with the P2Y12 receptor. The analgesic and anti-inflammatory effects of ICTF were completely reversed by administration of AM630, indicating that its actions are mediated via a CB2-dependent mechanism. This was additionally supported by molecular modelling suggesting that ICTF may bind to the CB2 orthosteric site [284].

Following intracerebral haemorrhage (ICH), thrombin accumulation induces cerebral oedema and blood–brain barrier (BBB) disruption, accompanied by microglial activation. In rats with thrombin introduced into the basal ganglia, activation of CB2 by JWH-133 (1.5 mg/kg i.p. starting 1 h after thrombin injection) significantly reduced the number of Iba-1-positive microglia in the region of thrombin injection and suppressed activation of the P44/P42 signalling pathway, which is commonly associated with microglial activation [285]. Furthermore, JWH-133 attenuated BBB permeability and decreased cerebral oedema. These protective effects were reversed by SR2.

Several studies have reported that modulation of CB2 activation in macrophages/microglia can alleviate neuronal damage and behavioural impairments following TBI, though one study instead indicated that inverse agonism is beneficial. In a controlled cortical impact (CCI) mouse model, CB2 agonist O-1966 (5 mg/kg i.p., 1–2/day for 1–4 days after CCI) attenuated blood–brain barrier disruption and neuronal degeneration, improved motor performance and exploratory behaviour, and suppressed expression of a macrophage/microglial activation marker as measured 1–7 days post-injury [286]. No inverse agonist competition study was undertaken. JWH-133 (1.5 mg/kg i.p., 1 h after injury) demonstrated efficacy in a CCI rat model by attenuating white matter injury and enhancing neurological function [263]. JWH-133 promoted the polarisation of primary microglia from an M1 to M2 phenotype. This transition promoted survival and maturation of oligodendrocytes, suppressing endoplasmic reticulum stress via inhibition of the protein kinase R-like endoplasmic reticulum kinase (PERK) signalling pathway, and upregulating phosphorylated Akt. These mechanisms contributed to the preservation of myelinated axons and functional recovery. All JWH-133 effects were blocked by SR2. Also in CCI mice, GP1a (3 mg/kg i.p., 10 min after injury) similarly promoted microglial polarisation from M1 to M2, leading to reduced cerebral oedema, improved cerebral blood flow, and enhanced neurobehavioural function [234]. Furthermore, there was a considerable increase in both CB2 gene expression and immunoreactivity following TBI. This was, at least in part, attributable to a marked infiltration of peripheral macrophages into the injury site, which was inhibited by GP1a. Interestingly, the specific dose of GP1a was important, with only 3 mg/kg producing maximal efficacy in all measures while the 1 mg/kg and 5 mg/kg doses varied from full to lack of efficacy depending on the outcome measure of interest. AM630 applied alone generally lacked effect on TBI outcome measures, except for reducing IL-6 gene expression similarly to GP1a [234]. These findings suggest that CB2 activation can beneficially regulate microglial responses across different TBI models, thereby contributing to neuroprotection and functional recovery.

Contrastingly, in a closed-head mild TBI mouse model, repeated administration of the CB2-selective inverse agonist SMM-189 (6 mg/kg/day i.p. for two weeks immediately after injury) promoted a phenotypic shift in microglia from M1 to M2, accompanied by significant improvements in motor, visual, and emotional functions [264]. A follow-up study with the same model and dosing found that SMM-189 enhanced nuclear pCREB in CNS microglia within 3 days of TBI and prevented ~50% neuronal loss as measured 2–3 months post-injury [287]. Cell-based studies on human and rodent primary microglia also support overall anti-inflammatory effects of SMM-189 on cytokine/chemokine secretion with presumed and neuroprotective consequences [129,264,288]. Interestingly, when monitoring M1 versus M2 polarisation in C8B4 mouse microglia stimulated with LPS (1 μg/mL), SMM-189 induced a phenotype that was seemingly intermediate between agonists JWH-133 and HU308, where expression of an M1 marker was reduced, and inverse agonist SR2, with increased expression of an M2 marker [129]. However, the cell-based studies used relatively high concentrations of SMM-189 (~10 μM) in comparison with its CB2 affinity (~120 nM) and functional potency (cAMP ~150 nM) [129], and none of these studies tested competition with other CB2 ligands or effects in CB2 KO animals or cells, despite SMM-189 having only ~40-fold binding selectivity over CB1. Interestingly, SMM-189 has been suggested to antagonise CP55,940 non-competitively [129]. To our knowledge, SMM-189 has not been evaluated when co-incubated with endocannabinoids, nor for impact on signalling pathways other than cAMP and inhibition of CP55,940-induced β-arrestin recruitment. Given the promising effects arising from this compound, it would be interesting to gain a better understanding of both these aspects to assist in interpreting the mechanism of action.

Post-traumatic trigeminal neuropathy is pain that occurs when the trigeminal nerve is damaged during oral and facial surgeries. Traditional pain relievers are ineffective against this pain which is caused by the activation of microglial cells in the caudal spinal trigeminal nucleus (Sp5C) region of the brain. In a mouse infraorbital nerve cut-induced cold hypersensitivity, HU308 (~12 μg/dose, four times over six days) inhibited microglial activation and reduced pain when administered intranasally [289]. This method was more effective than oral (p.o.) administration, and it was also confirmed that blocking CB2 with SR2 interferes with this effect.

Although distinct from neuropathic pain, chronic itch is also a sensory nerve-related painful condition. It causes severe discomfort for patients and initiates a vicious cycle in which scratching aggravates skin damage and inflammation. However, the CNS mechanisms of chronic itch remain unclear, and current clinical treatments are limited in efficacy. One study has focused on the role of stimulated immune cells in activating neuronal receptors and the regulatory function of CB2 in neuroimmune interactions [290]. Comparing a 1-fluoro-2,4-dinitrobenzene (DNFB)-induced chronic itch dermatitis/psoriasis model and a partial sciatic nerve ligation-induced chronic pain model (both mouse), AM1241 produced an anti-itch effect with significantly greater potency (ED_50_ 0.40 µg) than its analgesic effect (ED_50_ 3.26 µg). In mice with CB2 KO microglia, the analgesic effect was only partially reduced, whereas the anti-itch effect was significantly attenuated [290].

Further exploring the DNFB-induced model, CB2 expression was elevated in activated spinal dorsal horn microglia, as confirmed by immunostaining and mRNA analysis of primary microglia [290]. AM1241 (15 µg at various timepoints after DNFB induction) or GW405833 (30 µg, 12 days after DNFB induction) significantly suppressed scratching behaviour, an effect that was abolished by AM630. Furthermore, in CB2 KO mice, itch symptoms were exacerbated and AM1241 had no effect. Scratching behaviour was also exacerbated in mice lacking microglia, or with CB2 KO spinal microglia or peripheral macrophages, suggesting that microglial activation contributes to the condition. Furthermore, single-cell RNA sequencing revealed that AM1241 induced the anti-inflammatory regulator SOCS3 and inhibited phosphorylation of p38 and STAT1 in DNFB mice and similar outcomes were observed in IFN-γ-induced BV-2 cells, effectively reprogramming them toward an anti-inflammatory state. Lastly, AM1241 suppressed microglia-derived cytokines and reduced excitatory synaptic transmission in gastrin-releasing peptide (GRP)/GRP receptor interneurons and ascending projection neurons in DNFB-induced spinal cord. Investigating other itch models, AM1241 inhibited scratching behaviour in the imiquimod-induced model, another chronic itch paradigm, but had no effect in three acute itch models. Overall, these findings support that microglia activation in spinal cord contributes to chronic itch and that CB2 activation by AM1241 leads to anti-pruritic effects by modulating neuroimmune interactions [290].

### 6.3. Neuroinflammatory and Neurodegenerative Disorders

CB2 has been shown to play a regulatory role in neuroinflammatory and neurodegenerative diseases such as Alzheimer’s disease (AD), Huntington’s disease (HD), Parkinson’s disease (PD), and amyotrophic lateral sclerosis (ALS). CB2 agonists tend to attenuate inflammatory responses and reduce neuronal damage. Autoimmune disease multiple sclerosis (MS) also involves neuroinflammation and neurodegeneration and is explored in Section 6.4.

The potential for CB2 modulation to influence AD progression and pathology has been investigated in a range of animal models. The two major pathological features of AD are amyloid plaques and neurofibrillary tangles (NFTs). Amyloid plaques are lesions formed by the accumulation of amyloid-β (Aβ) peptides, which are generated through the cleavage of amyloid precursor protein (APP), outside of neurons. Plaque detection by PET or in cerebrospinal fluid can be utilised as a diagnostic biomarker indicating the early pathological stage. NFTs are formed by the abnormal intracellular accumulation of hyperphosphorylated tau protein, the presence of which is considered a progressive biomarker that correlate with disease severity, prognosis, and cognitive decline. In particular, the spread of tau pathology is closely associated with neuronal damage and cognitive impairment [291].

CB2-selective agonists have demonstrated potential for neuroprotective effects in human cell and rodent AD models. Low concentrations of JWH-015 (1–100 nM; maximum effect at 10 nM) stimulated THP-1 macrophages (differentiated with PMA) to clear Aβ from AD patient brain tissue sections [292]. This effect was blocked by SR2, supporting that it was mediated through CB2. JWH-015 (10 nM) also stimulated phagocytosis of pure fluorescently labelled Aβ by THP-1 macrophages, but this was not reversed by SR2. In an APP/PS1 mouse study, results for JWH-015 were mixed. Although JWH-015 (0.5 mg/kg/day i.p. for 8 weeks from 8 months old) improved novel object recognition and reduced microglial activation markers, there was no change in spatial memory performance or amyloid plaque burden [260]. More substantial outcomes were obtained in mutated APP (long form of human APP with double mutation) transgenic mice, where oral administration of JWH-133 (0.2 mg/kg/day p.o. aq. ad lib. for 4 months from 7 months of age) improved cognitive deficits, suppressed inflammation, restored brain metabolic activity, and normalised the elevated density of Iba1-positive microglia [293]. However, supporting evidence with a CB2 antagonist was not presented. In rats injected with Aβ fibrils into the hippocampus, MDA7 (15 mg/kg i.p. daily for 14 days from first Aβ injection) also reduced inflammatory markers, promoted Aβ clearance, and reversed symptoms of cognitive decline [294]. In the behavioural test, the effect of MDA7 was reversed by AM630.

While pharmacological activation of CB2 has consistently demonstrated protective effects in various disease models, results from CB2 KO in conjunction with disease models have been more variable, potentially due to differences in the stage of disease progression and the specific AD model utilised. Aβ plaque burden and total level of tau proteins were measured in a 12-month-old J20 mouse model that expressed human mutant APP in combination with CB2 KO [295]. Plaque burden was significantly increased in CB2 KO mice. While there was no difference in the overall activation of microglia based on Iba-1 staining, microglia closely associated with the plaques were more abundant in the CB2 KO group. Interestingly, total tau protein levels were significantly lower in the CB2 KO mice. Although these findings suggested that CB2 plays an important role in suppressing Aβ plaque pathology, CB2 activation may have an unexpectedly deleterious effect on tau pathology, suggesting that CB2-targeted therapies may produce opposing outcomes depending on the pathological stage [295]. Contrasting findings were observed in APP/PS1 mice. Genetic deletion of CB2 led to overall anti-inflammatory effects, with a marked reduction in the proportion of microglia and infiltrating macrophages in the brain, along with decreased production of pro-inflammatory chemokines and cytokines such as CCL2, IL-6, and TNF [235]. Soluble Aβ levels were reduced at 9 months of age, and at 14 months of age a corresponding decrease in Aβ plaque burden was detected, though the concentration of soluble Aβ in brain homogenates was equivalent with or without CB2 KO. 6-month-old CB2^−/−^ mice, with or without APP/PS1, exhibited significantly less impaired performance in a spatial memory task compared to animals with intact CB2. This improvement was not evident in mice aged 9- or 14-month-old, again, with or without APP/PS1 [235]. A subsequent study on APP/PS1 × CB2^−/−^ mice implemented more rigorous experimental conditions in the spatial memory test by extending the intertrial time [296]. Deletion of CB2 enhanced cognitive performance, but this time, only for 14-month-old mice with no difference at 9 months. As in the prior study, at 14 months amyloid plaque accumulation was decreased, and CB2 KO reduced neuronal loss in the cortex (with an equivalent trend but no significant difference in the hippocampus). The overall findings in APP/PS1 mice contrast with the prevailing view that activation of CB2 provides anti-inflammatory and neuroprotective benefits. It is worth considering that the age-dependent differences observed in CB2 KO mice may implicate adaptive and compensatory mechanisms in response to CB2 deficiency in the effects on AD-related outcomes. Supporting evidence from other research involving CB2-deficient mice also aligns with this interpretation [297]. Follow-up studies from the same lab provided insights that help to explain these unexpected results. One found that acute pharmacological inhibition of CB2 with SR2 failed to reproduce the anti-inflammatory effects observed in CB2 KO mice, except at high concentrations likely to have caused non-CB2-mediated effects [298]. Another demonstrated that chronic CB2 deficiency reduced the transcriptional response of microglia to TLR stimulation, mainly through the p38 MAPK signalling pathway, thus reducing the responsiveness of CB2 KO microglia to inflammatory stimuli [299]. Taken together, these findings indicate that the genetic deletion of CB2 may induce developmental or homeostatic adaptations beyond simple receptor inhibition.

In HD patients and mouse models, observations of increased CB2 expression in microglia (Section 4.2, [181]) motivated examination of the consequences of CB2 KO on HD-related pathology and symptoms. CB2 gene deletion enhanced microglial activation, aggravated disease symptoms, and ultimately shortened the lifespan of mice lesioned with intrastriatal quinolinic acid (QA) [181]. In contrast, HU308 (5 mg/kg/day i.p. starting after QA lesion) reduced neuroinflammation, brain oedema, neuronal damage, and motor symptoms.

Parkinson’s disease (PD) is characterised by the selective degeneration of dopaminergic neurons. In an MPTP-induced PD mouse model, JWH-133 (10 μg/kg/day i.p., starting two days prior to MPTP and for 8 days after) exhibited neuroprotective effects by preventing the degeneration of dopaminergic neurons [300]. This effect was associated with the inhibition of peripheral immune cell infiltration and the suppression of inducible nitric oxide synthase (iNOS), pro-inflammatory cytokines, and chemokines produced by activated microglia. A similar neuroprotective profile was observed with the non-selective cannabinoid receptor agonist WIN55,212 (same dosing as JWH-133), whereas co-administration of AM630 abolished both the neuroprotection and suppression of glial-mediated neurotoxicity [300].

In ALS mouse models SOD1-G93A [301] and TDP-43-A315T [302], administration of a CB2 agonist (AM1241 0.3 or 3 mg/kg/day i.p. [301], or HU308 5 mg/kg/day i.p. [302]) from symptom onset prolonged survival in the AM1241 study and improved motor function and contributed to the preservation of motor neurons in the HU308 study. In the latter study, similar effects were observed with WIN55,212-2 which were blocked by AM630. However, contrasting effects of CB2 activation have been reported in the SOD1-G93A model. Although CB2 agonists (three relatively novel RO compounds, 10 mg/kg/day i.p. from ALS gene induction) suppressed microglial expansion and activation markers, this induced acute detrimental effects on levels of misfolded SOD1, whereas a CB2 inverse agonist had the opposite effect [303]. A distinction in this study was that treatment was initiated pre-symptomatically. While effects on ultimate symptomology and survival were not investigated, this study raises the possibility that the timing of treatment may be impactful as to whether it is optimal to activate versus inhibit CB2 in potential ALS treatment.

Epilepsy is characterised by abnormal neuronal firing that manifests in seizures, may arise from various causes including brain injury and genetic mutations, and is modulated by neuroinflammation. Cannabinoids have received considerable attention for their potential to regulate seizures, usually with focus on CB1 modulation of neuronal activity, and a particular success is CBD, which has been approved for treating severe epilepsy syndromes (see also Section 7.1). CB2 has undergone comparatively lesser investigation, but the potential for CB2 to regulate epilepsy via neuronal, astrocytic, or microglial activity is recognised [304]. CB2 inverse agonism or KO has been reported to increase seizure susceptibility [305,306]. Two studies have specifically reported data regarding the role of microglial CB2 in epilepsy models. AM1241 (1 or 3 mg/kg/day i.p. from 8th day after induction of status epilepticus) suppressed seizure frequency in a pilocarpine-induced chronic epilepsy mouse model, though duration and severity were unchanged [216]. This was accompanied by decreased neuronal loss and depressive-like behaviour. AM630 applied alone (1 mg/kg i.p., same dosing schedule) had no significant effects. As detailed in Section 6.2, AM1241 also reduced inflammasome activation and other inflammatory markers in hippocampal microglia. Furthermore, CB2 but not CB1 mRNA increased in the hippocampus post-status epilepticus, and protein expression correlated with activated microglia. In a kainite-induced status epilepticus mouse model, CB2 mRNA was again slightly upregulated in the hippocampus whereas CB1 expression decreased [288]. However, CB2 inverse agonist SMM-189 (6 mg/kg i.p. twice daily, after stopping 1 h of status epilepticus) was anti-inflammatory and neuroprotective, improving functional recovery, reducing levels of pro-inflammatory cytokines, and reducing activated microglia marker Iba1 and astrocyte marker GFAP mRNA in the hippocampus. This finding that CB2 blockade is beneficial in epilepsy and suppressed microglial activation is in apparent contrast to conclusions from most other studies to date. It is unknown whether this is a model or treatment timing-related difference or might be specific to the SMM-189 ligand given unexpected effects have also been observed in other contexts (see also Section 6.2).

### 6.4. Autoimmune Diseases

Autoimmune disease progression primarily involves B and T cell responses. However, the roles of macrophages in autoimmune diseases have also received increasing attention. Infiltration of macrophages is elevated in autoimmune lesions, and these macrophages tend to be hyperactivated as a result of imbalance between the M1 and M2 phenotypes (see also Section 2.3). Several studies on autoimmune diseases have provided insight into the effects of CB2 activation on macrophages infiltration and activity in this context.

Extensive research on MS has been undertaken using EAE or TMEV animal models, with a number of studies indicating therapeutic benefit from CB2 activation. In the EAE model, CB2 KO exacerbated disease severity and was associated with increased microglial activation, T cell infiltration, and axonal loss from neurons [179]. Further supporting an active role for CB2, HU308 (15 mg/kg i.p. daily, starting after observation of robust MS-related symptoms) reduced symptoms and associated inflammatory markers, with no such effect in CB2 KO animals [179]. Subsequently, concentration-dependence of the HU308 effect was demonstrated with 0.3 mg/kg/day having no significant effect, but 1 and 3 mg/kg/day (i.p., starting from day 3 after EAE induction) reducing EAE severity score and the highest dose also reducing leukocyte infiltration into the spinal cord and extent of demyelination [215]. BCP (5 [261], 25, and 50 [307] mg/kg p.o.) can also reduce symptom severity and associated inflammatory markers in the EAE model, as can dual CB2/PPARγ agonist VCE-004.8 (10 mg/kg i.p.) (also known as Etrinabdione, see Section 7.2 and Table 3) in two models of MS, EAE, and TMEV [308]. Indirect approaches to CB2-mediated anti-inflammatory effects have also been tested by enhancing endocannabinoid levels via inhibition of uptake or degradation. In the TMEV model, AEA uptake inhibitor UCM707 (5 mg/kg/day i.p. for 12 days starting after onset of symptoms) restored motor function and reduced inflammatory markers, though the specific mediator of these effects was not verified [309]. In the EAE model, treatment with the ABHD6 inhibitor WWL70 (5 and 10 mg/kg/day i.p. from symptom onset) increased 2-AG levels and reduced inflammation, including microglial activation, and neurodegeneration [310]. These effects were abolished by co-administration of AM630 (3 mg/kg i.p.) (but not CB1 inverse agonist AM281), and with CB2 KO symptoms were exacerbated with no impact of WWL70 treatment, supporting that the anti-inflammatory and neuroprotective actions of WWL70 were CB2-mediated [310].

SSc is an autoimmune disease in which fibroblast dysfunction leads to abnormal collagen accumulation and progressive fibrosis in the skin and internal organs. The anti-fibrotic effect of VCE-004.8 was evaluated in a bleomycin (BLM) induced SSc mouse model (BLM subcutaneous injections for 6 weeks, with 10 or 20 mg/kg VCE-004.8 in the last 3 weeks of BLM injections) [311]. While mice treated with BLM for 6 weeks showed a significant increase in dermal thickness and collagen accumulation, skin fibrosis was attenuated by treatment with VCE-004.8. Pre-treatment with AM630 or the PPARγ antagonist, T007907, partially abrogated the effect of VCE-004.8, suggesting that its full anti-fibrotic effect depends on activation of both PPARγ and CB2. Furthermore, skin biopsies exhibited significantly increased infiltration of F4/80(+) macrophages with BLM injection, which was suppressed by VCE-004.8 (20 mg/kg) or rosiglitazone (PPARγ agonist) [311]. In another study using BLM-induced SSc mice, the anti-fibrotic effect of VCE-004.8 was similarly observed when administered as an oral lipid formulation (EHP-101) [312]. Both skin fibrosis and macrophage infiltration were reduced after daily oral gavage of EHP-101 (5, 10, and 25 mg/kg) during the last three weeks of BLM injection. In a follow-up study, Arg-1 expression was compared between groups receiving daily oral gavage of 20 mg/kg EHP-101 or 5 mg/kg ajulemic acid (AJA, also known as lenabasum) [313]. Arg-1 expression was reduced by EHP-101, but not by AJA. This finding was interpreted to imply that EHP-101 inhibited M2 macrophage activation as Arg-1 is a M2 macrophage marker. However, this Arg-1 expression cannot be conclusively attributed to being of macrophage origin because the analysis did not include double staining with a macrophage marker and Arg-1 is expressed in other immune cells as well as upregulated in fibroblasts and keratinocytes in some pathological conditions.

### 6.5. Non-Neuronal Tissue Injury and Chronic Inflammation

Suppression of inflammatory responses and fibrosis induced by CB2 activation has been reported in non-neuronal organs including the liver, intestine, and lungs, as well as in various tissues such as ocular tissue, vasculature, and muscle.

The regulatory function of CB2 has been reported in both acute and chronic liver inflammation and injury. In a concanavalin A-induced acute liver injury model, CB2 KO mice exhibited significantly increased expression of macrophage markers CD68 and F4/80, as well as the pro-inflammatory cytokine TNF [314]. These mice also showed elevated serum alanine aminotransferase (ALT) levels and more severe hepatic tissue damage, indicating that CB2 is involved in suppressing excessive macrophage activation and thereby mitigating inflammatory responses in the liver. Moreover, in two cirrhotic ascitic rat models (thioacetamide and bile duct ligation), JWH-133 (1 mg/kg/day by oral gavage, for 2 weeks after establishing stable ascites) reduced oxidative stress and inflammation both systemically and in the gut, suppressed TNF secretion, and alleviated bacterial translocation, spontaneous bacterial peritonitis, intestinal mucosal damage, and increased permeability [205]. JWH-133 also enhanced the expression of intestinal tight junction proteins and inhibited TNF receptor and NF-κB p65 protein expression. In peritoneal macrophages isolated from dosed cirrhotic rats, JWH-133 restored TNF-suppressed phagocytosis. JWH-133 effects were reversed by co-administration of AM630, while ACEA alone had no effect [205]. Meanwhile, in chronic alcohol-induced liver injury mice, BCP (10 mg/kg/day i.p., starting with alcohol feeding) produced anti-inflammatory effects, inhibiting the conversion of Kupffer cells to a pro-inflammatory M1 phenotype (as is usually observed during the course of alcohol feeding), and reducing the expression of vascular adhesion molecules ICAM-1, E-selectin, and P-selectin [315]. Furthermore, BCP positively influenced hepatic metabolic dysregulation, including steatosis, protein hyperacetylation, and PPAR-α signalling. These protective effects of BCP were diminished in CB2 KO mice, suggesting that its hepatoprotective action involves CB2 activation.

Proliferative vitreoretinopathy (PVR), a common complication that can arise following ocular trauma, inflammation, or retinal detachment surgery, currently has no available pharmacological treatment. In a PVR model (induced by dispase injection), pathological changes in the eye were compared between CB2 KO and WT mice [316]. CB2 KO mice exhibited aggravated symptoms and enhanced inflammatory responses, as indicated by elevated cytokine protein levels and increased microglial infiltration. In WT mice, HU308 (1 mg/kg/day i.p. for 7 days following PVR induction) improved pathological symptoms and reduced the number of microglia in retinal section with PVR. These effects were reversed by pre-treatment with AM630. In contrast, AM630 alone (2.5 mg/kg/day i.p. for 7 days) exacerbated pathological features, but this deterioration was not alleviated by HU308 pre-incubation [316].

Macrophage apoptosis is a pathological mechanism involved in atherosclerosis. The accumulation of apoptotic macrophages exacerbates inflammation and tissue damage within lesions, contributing to plaque instability and increasing the risk of cardiovascular events. Macrophages from CB2 KO mice exhibited significantly lower levels of apoptosis than wild-type cells following exposure to oxLDL or 7-ketocholesterol (7KC), a major oxysterol component of oxLDL for 16 h [317]. This was accompanied by reduced caspase-3 activity, suppressed PARP cleavage, and impaired inactivation of the pro-survival kinase Akt. These findings suggest that CB2 facilitates oxysterol-induced apoptosis in macrophages by modulating the Akt pathway [317]. Meanwhile, pre-treatment with JWH-133 (0.1–10 μM) or HU308 (10 μM) enhanced the ability of RAW264.7 and mouse primary macrophages to efferocytose UV-induced apoptotic RAW264.7 cells [206]. Presence of oxLDL for 45 min impaired efferocytosis, but this was reversed by CB2 stimulation in a concentration-dependent manner (both agonists effective at 0.1–10 μM for primary macrophages). CB2 activation also increased the expression of phagocytic receptors of the tyrosine kinase family, reduced oxLDL-induced production of TNF and ROS, and inhibited RhoA GTPase activation, thereby attenuating inflammatory signalling [206]. Collectively, these results suggest that CB2 may play a dual role in atherosclerosis by modulating both macrophage apoptosis and efferocytosis, ultimately influencing the inflammatory microenvironment and plaque stability.

Skeletal muscle is particularly susceptible to external injury, and the healing process of damaged muscle typically begins with an inflammation-driven response. MAGL is a key enzyme responsible for degrading 2-AG, thereby regulating cannabinoid receptor activity. JZL184 is a selective MAGL inhibitor that increases intracellular levels of 2-AG by preventing its degradation, indirectly enhancing cannabinoid receptor signalling. JZL184 has previously been shown to reduce inflammation in colitis and acute lung injury mouse models. In a rat muscle contusion model, JZL184 (10 mg/kg/day i.p. for 5 days after contusion) significantly reduced neutrophil and macrophage infiltration, as well as the expression of pro-inflammatory cytokines [318]. However, as the post-injury interval progressed, myofibre regeneration was markedly impaired, accompanied by increased mRNA expression of collagen, fibroblast infiltration, and enhanced fibrosis. Interpretation of inverse agonist experiments was complex, as AM630 had some anti-inflammatory effects when applied alone. However, where AM630 did not have an effect alone, AM630 reversed all tested anti-inflammatory effects of JZL184, whereas CB1 inverse agonist AM251 only reversed IL-1β suppression, suggesting that the majority of these effects are primarily mediated via CB2 [318].

### 6.6. Cancer and Tumour Microenvironment

Glioblastoma, an aggressive brain cancer, is characterised by a highly immunosuppressive tumour microenvironment and systemic immunosuppression. Bioinformatic analysis of human glioblastoma patient databases revealed that overexpression of CB2 is associated with both poor clinical outcomes and immune-related signalling pathways [266]. DAGLB and ABHD6, 2-AG synthesising and degrading enzymes, were negatively and positively correlated with survival, respectively [266], while MAGL KO was associated with enhanced TAM acquisition of an M2 phenotype and promotion of tumour progression (see also Section 5.5) [266].

In a murine model implanted with GL261 glioblastoma cells, treatment with AM630 (5 mg/kg i.p., every second day for two weeks from 1 week after cell implantation) suppressed tumour growth and significantly prolonged survival [266]. The AM630-treated group exhibited a 50% survival rate at day 40, at which point mortality in the control group reached 100%, whereas control and CB2 agonist-treated mice died earlier (GW405833, 5 mg/kg i.p., same dosing schedule as AM630). Immune cell profiling showed that AM630 treatment reduced monocyte/macrophage populations in the spleen, while CD8^+^ and CD4^+^ T cell populations increased. Within tumours, TAMs decreased while CD8^+^ T cells tended to increase. Furthermore, in in vitro co-culture experiments, lymphocytes from AM630-treated mice exhibited enhanced cytotoxic function, as evidenced by increased proportions of perforin- and granzyme B-positive CD8^+^ T cells [266]. CB2 siRNA knockdown or AM630 treatment also reduced secretion of TAM-associated cytokines in human MDMs exposed to apoptotic cancer-conditioned medium (see also Section 5.4) [250]. These results indicate that CB2 activation contributes to immune evasion in glioblastoma, while CB2 inhibition may restore antitumour immunity.

Despite these positive effects of CB2 KO or blockade, it is important to keep in mind that CB2 is expressed on some tumour cells [319]. In these cases, the potential impact of blocking CB2 on tumour cell growth should be considered. For example, in a non-small cell lung cancer model, CB2 activation with JWH-015 produced beneficial effects by inhibiting the acquisition of a malignant phenotype and reduced tumour growth (effects were blocked by SR2) [320]. However, outcomes of CB2 activation or blockade may well depend on the specific cancer and/or stage, implying consideration to the responses of all involved cell types is needed when considering CB2-targeted therapeutic design in this context [319].

## 7. Clinical Evaluation of Cannabinoids

Preclinical research on medical cannabis and cannabis-derived compounds has demonstrated significant potential in the treatment of various immune-related disorders, including some types of pain, inflammatory bowel disease (IBD), rheumatic disease, atherosclerosis, and atopic dermatitis, primarily through CB2 activation [95,197,321]. These compounds also possess potential to be therapeutically useful in neuroinflammation and neurodegenerative disorders, in part because cannabinoids typically have high lipophilicity and can cross the blood–brain barrier (BBB) [322,323]. However, the ability to penetrate the BBB simultaneously raises concerns about potential psychoactive effects mediated by CB1 activation. Although some non-CB1/CB2-selective, CNS-penetrant, cannabinoids have been found to be effective and are approved for specific indications (Section 7.1, Table 2), limiting cannabinoids to the periphery and/or selectively targeting CB2 as a potent immune modulator is attractive to avoid psychoactive effects [113]. Indeed, compounds selectively targeting CB2, and increasingly with optimised physicochemical properties, have been developed and a selection evaluated in clinical trial (Section 7.2, Table 3).

### 7.1. Cannabis-Derived Compounds

Before the 2000s, pharmaceutical preparations of synthetic THC, dronabinol (Marinol^®^ and Syndros^®^) and a THC analogue nabilone (Cesamet^®^), were approved by the USA Food and Drug Administration (FDA) as treatments for cancer chemotherapy-induced side effects, such as nausea and vomiting, or HIV/AIDS-induced anorexia [324]. Numerous clinical trials on medical cannabis-derived drugs have been conducted subsequently, leading to a handful of further approvals (Table 2). A well-established example is Nabiximols (Sativex), containing cannabis-derived THC and CBD, which has received approval in over 27 countries for managing MS symptoms due to production of significant improvement in muscle spasms, a key symptom of MS [80,324]. Another medicinal cannabis product, Epidiolex, a CBD oral solution, was approved by the FDA in 2018 for treating rare childhood epilepsies, specifically Lennox–Gastaut syndrome, Dravet syndrome, or tuberous sclerosis complex [80,325].

**Table 2:** FDA and/or international regulatory body-approved cannabis-based pharmaceuticals [80,326].

Drug Name	Description	Approved Uses	Earliest Approval Year
Dronabinol (Marinol^®^)	Synthetic THC (tetrahydrocannabinol)	Chemotherapy-induced nausea and vomiting (CINV), HIV/AIDS-induced anorexia	1985 (CINV), 1992 (HIV/AIDS)
Nabilone (Cesamet^®^)	Synthetic cannabinoid, THC analogue	Chemotherapy-induced nausea and vomiting	1985
Nabiximols (Sativex^®^)	Cannabis-derived THC and CBD combination	Multiple sclerosis (MS)-related muscle spasticity and neuropathic pain	2010
Syndros^®^	Oral solution form of Dronabinol	Chemotherapy-induced nausea and vomiting, HIV/AIDS-induced anorexia	2016
Epidiolex^®^	Purified CBD oral solution	Epilepsy (Lennox–Gastaut syndrome, Dravet syndrome, tuberous sclerosis complex)	2018

At present, a number of clinical trials around the world are investigating the effects of medical cannabis treatments on various diseases are underway and progressing toward regulatory approval.

Patients with IBD, which includes Crohn’s disease (CD) and ulcerative colitis (UC), often turn to cannabis-derived compounds to alleviate symptoms, such as abdominal pain, diarrhoea, and nausea instead of using corticosteroids and other immunosuppressors which have drawbacks to use including that long-term use of these medications have been associated with complications, such as malignancy and infection [327,328]. The pathophysiology of IBD is characterised by excessive intestinal inflammation and dysregulation of immune homeostasis, which is mainly controlled by macrophages [329]. CD is known to be associated with upregulated Th1 cytokines, such as IL-12, IFN-γ, and TNF, while UC is associated with a Th2 profile induced by innate immune cells such as macrophages. Despite differences in cytokine profiles between CD and UC, the key to treating IBD is modulating increased pro-inflammatory cytokine production derived from both innate and adaptive immune cells and enhancing the recruitment of immune cells, which may be achieved by using medicinal cannabis [100,330]. Various clinical studies have been conducted to evaluate the efficacy of cannabis in treating IBD. These studies showed promise of various formulation of medical cannabis including smoked cannabis, cannabinoid oil and cannabidiol capsules in improving quality of life and symptoms, although further studies are still required to demonstrate statistical significance to prove efficacy [331].

Many patients with skin disorders, such as psoriasis and atopic dermatitis, are being prescribed various formulations of cannabis products, such as topical CBD, oral dronabinol, and oral hempseed oil, to support conventional treatment. Although prescribed as off-label for these conditions, regulatory approval is still pending [332]. These skin disorders, such as psoriasis, involve dysregulation of the immune response by macrophages [333], hyperproliferation and increased infiltration of immune cells in the skin. Therefore, the anti-inflammatory effects of cannabis compounds are considered beneficial for these inflammatory skin disorders. In clinical trials, the effects of medical cannabis on the skin disorders have showed promise of CBD with hemp oil and CBD ointment in improving quality of life, skin elasticity and severe itching. However, further clinical evidence, including randomised trials with larger numbers of participants, is required to further validate efficacy and establish standards for dosage and administration methods for medical cannabis [332,334,335].

### 7.2. CB2-Selective Compounds

Numerous CB2-selective agonists and inverse agonists have been developed and are currently in various stages of characterisation [113,115,336]. However, to date, only fourteen have reached clinical trial, with just one advanced to phase 3 trial (Table 3). Trial outcomes have been mixed; although multiple compounds indicated lack of efficacy for the indication selected for trial, a few compounds had significant effects on primary or secondary outcome measures, or efficacy indicated in a specific participant subgroup. As well, the class has generally been found to be safe, with nearly all trials reporting lack of notable adverse effects. While the majority of completed trials for CB2-targeted agonists have focused on various types of pain, with a few studying cystic fibrosis or autoimmune disorders, at writing trials have been initiated for Alzheimer’s disease (AD), arterial disease, and cancer anorexia, which are previously unexplored indications. Table 3 provides an overview of CB2-targeted compound clinical trials to date. Selected examples where the mechanism of action relates to modulating macrophage activity are discussed below.

**Table 3:** Status and outcomes of CB2-selective drug clinical trials.

Drug Name °	CB2 > CB1 Selectivity [113,115] ^■^	Indication	Phase Complete	Reported Outcome	Year Reported	Sponsor
Lenabasum *	12 ^Δ^	Neuropathic Pain	2	Significant reduction in pain score vs. placebo [337].	2003	Atlantic Tech./Indevus Pharm.
Cannabinor (PRS-211,375)	~320 ^◆^	Nociceptive Pain	2	Reduced pain vs. placebo at lowest but not two higher doses [338].	2007	Pharmos
GW842166	>270 ^◆^	Osteoarthritis Pain	2	No significant pain relief compared to placebo [339].	2008	Glaxo-SmithKline
S-777469	~130	Atopic Dermatitis	2	No significant effect on physician’s global assessment compared to placebo [340,341].	2009	Shionogi
Tedalinab (GRC 10693)	>4630 ^◆^	Neuropathic/Inflammatory Pain	1	No significant or serious adverse events [342].	2009	Glenmark Pharmaceuticals
GW842166	>270 ^◆^	Post-surgical Pain	2	No significant pain relief compared to placebo [343].	2011	Glaxo-SmithKline
AZD-1940 (ART-27.13)	13 ^⯌^	Post-surgical/Acute Pain	2	No effects on pain scores in two trials. Adverse events consistent with CNS activity [344,345].	2013	AstraZeneca
KHK6188	(not disclosed)	Neuropathic Pain	2	Highest dose effective in relieving postherpetic neuralgia [346].	2015	Kyowa Hakko Kirin
LY2828360	32 ^◆^	Osteoarthritis Pain	2	No significant change in primary pain score. Potential effects on two exploratory pain models [347,348].	2020	Eli Lilly
Etrinabdione (EHP-101, VCE-004.8)	>230 ^Δ^	Multiple (MS) and Systemic Sclerosis (SSc)	1 (2)	Two phase 2 trials initiated but suspended for commercial reasons [349,350].	2020	Emerald Health
Lenabasum *	0.1–33 ^Δ,⯌^ [351]	Cystic Fibrosis	2	2a: No change in forced expiratory volume, but reduced inflammatory markers at highest dose [352]. 2b: Pulmonary exacerbation primary/secondary endpoints not met [353,354].	2021	Corbus Pharmaceuticals
Lenabasum *	0.1–33 ^Δ,⯌^ [351]	Systemic Lupus Erythematosus (SLE)	2	No significant differences in pain or disease activity outcome measures [355].	2022	National Inst. Allergy & Infectious Diseases
Lenabasum *	0.1–33 ^Δ,⯌^ [351]	Dermatomyositis	3	Did not meet primary or secondary endpoints, but decreased inflammatory markers [356,357]. Improvement in muscle strength and rash in subgroup analysis.	2023	Corbus Pharmaceuticals
Lenabasum *	0.1–33 ^Δ,⯌^ [351]	Systemic Sclerosis (SSc)	3	With background immunosuppressive treatment (IST), no significant effect on CRISS score. Trend toward improvement vs. placebo without IST [358].	2023	Corbus Pharmaceuticals
Olorinab (APD371)	>1200 [359]	Crohn’s Disease (CD), Irritable Bowel Syndrome (IBS)	2	2a (CD): Reduced abdominal pain over time (not placebo controlled) [360]. 2b (IBS): Primary endpoint not met. Subgroup with pain score improvement vs. placebo [361].	2023	Arena Pharmaceuticals
NTRX-07	27	Alzheimer’s Disease (AD)	1 (2)	No dose-limiting or serious adverse events. Trend toward improved cognitive scores in AD patients [362,363]. Phase 2 recruiting [364].	2023	NeuroTherapia
CNTX-6016	>15,000 [365]	Diabetic Neuropathy	1	1a completed 2019, 1b completed 2023; results not disclosed [366,367].	(2023)	Centrexion Therapeutics
TT-816 °	>380 [368]	Cancer (solid tumours)	(1 + 2)	Phase 1/2 trial initiated but suspended for commercial reasons [369].	(2023)	Teon Therapeutics
Vicasinabin (RG7774)	>195 ^◆^	Diabetic Retinopathy	2	No significant effect on retinopathy progression vs. placebo [370].	2024	Hoffmann-La Roche
AZD-1940	13 ^⯌^	Cancer Anorexia	(1 + 2)	Trial recruiting [371].	(2025)	Artelo Biosciences
EHP-101	>230 ^Δ^	Arterial Disease	(2)	Upcoming trial registered [372].	(2025)	VivaCell Biotechnology España

Drug list informed by recent reviews [113,115,336]. Trial data and status based on clinical trial databases (at May 2025; ClinicalTrials.gov [373] and WHO ICTRP [374]) and as indicated in table. ° All drugs in Table are CB2 agonists, except TT-816 which is an inverse agonist [375]. ^■^ Fold selectivity for CB2 > CB1, based on binding affinity, except ^◆^ based on functional potency. Drugs have not necessarily been tested for affinity/efficacy at targets other than CB2 and CB1. ^Δ^ Etrinabdione and lenabasum are also PPARγ agonists; selectivity CB2 > PPARγ: Etrinabdione ~10 [311], Lenabasum ~3.5–14 [351,376]. * Lenabasum is also known as ajulemic acid, anabasum, CPL7075, CT-3, HU-239, IP751, JBT-101, O-981-6, Resunab. ^⯌^ Lenabasum and AZD-1940 have low CB2 > CB1 selectivity but are suggested to have limited CNS permeability; peripheral CB1 activation may contribute to mechanism of action [344,377].

Lenabasum, also known as JBT-101 and many other names (see Table 3 *), has a similar structure to a THC metabolite. Lenabasum is a mixed CB2/CB1 agonist, with only modest selectivity toward CB2 and the specific degree of selectivity dependent on the method/purity of compound preparation [351]. Preclinical trials found limited blood–brain-barrier permeability indicating that psychoactivity via central CB1 receptors may be avoided, though activation of peripheral CB1 might contribute to lenabasum’s mechanism of action [378]. Lenabasum can also activate nuclear receptor PPARγ, which is itself a potential anti-inflammatory target [379]. Therefore, lenabasum’s effects may well involve polypharmacology and only partially be reliant on CB2. Despite this potential for complex mechanism of action, lenabasum is particularly notable as the most thoroughly characterised CB2-active compound in human trials to date, including in diseases likely involving macrophage dysregulation, cystic fibrosis, systemic sclerosis (SSc), and dermatomyositis.

Cystic fibrosis is a condition characterised by chronic airway inflammation, which may be driven by macrophage activation and excessive cytokine release, leading to the destruction of lung tissue and lung dysfunction [380]. Since pulmonary inflammation is a significant contributor in the pathophysiology of cystic fibrosis, patients have been prescribed immunosuppressants which have notable adverse effect profiles [381,382,383]. In a double-blind, randomised, and placebo-controlled phase 2 trial of 89 cystic fibrosis patients, 51 participants completed 8–12 weeks of lenabasum treatment, with three discontinuing due to treatment-related adverse events (versus 23 completed placebo, one placebo treatment-relate withdrawal) [352]. Although there was no significant difference in the percent predicted forced expiratory volume between groups, lenabasum showed potential in reducing inflammatory markers, such as IL-8, IgG, and inflammatory cell infiltration at a 20 mg twice-a-day dose subgroup. This was followed by a larger phase 2 study with over 400 participants for 28 weeks, with subsequent safety follow-up [353]. Primary and secondary efficacy endpoints relating to pulmonary exacerbations were not met, though the drug was well tolerated. To further elucidate the mechanisms of lenabasum’s clinical effects in macrophages, functional activity, and polarisation were explored in MDMs from cystic fibrosis patients [204]. Lenabasum suppressed M1 polarisation and the secretion of IL-8 and TNF in a dose-dependent manner and enhanced phagocytic activity, while it did not restore impaired M2 polarisation. These effects were not observed in macrophages in healthy donors.

SSc is a rare autoimmune disorder characterised by chronic inflammation, fibrosis, and vessel damage in tissues driven by the overactivation of the innate immune system including macrophages [384]. Immunosuppressants like glucocorticoids have been prescribed to suppress the immune response; however, these drugs pose risk to organs, such as glucocorticoid-induced renal crisis [383]. A phase 2 study investigated the therapeutic effects of lenabasum on SSc in 42 subjects (27 drug, 15 placebo) through a randomised, double-blind, placebo-controlled trial [385]. 90% of study participants were receiving background immunosuppressive medications. The study reported therapeutic improvements in overall efficacy assessments, such as the American College of Rheumatology Combined Response Index in diffuse cutaneous SSc (CRISS) score, skin involvement and patient-reported function with between 8 and 16 weeks of lenabasum treatment. In contrast, a phase 3 trial for SSc with 365 participants randomised to two doses of lenabasum or placebo for 52 weeks did not meet its primary endpoint, with no difference identified between the placebo and treatment group in CRISS score or skin thickness score [358]. All groups, regardless of lenabasum treatment, showed significant symptom improvement compared to before treatment, which was attributed to the patients continuing background immunosuppressive medications. In comparison with the earlier phase 2 trial, a wider range of immunosuppressive medications and a shorter minimum time on the background medication were permitted. It was surmised that in the phase 3 trial the disease was suppressed to a degree where a ceiling effect was reached and potential additional effects of co-administered lenabasum were not observable. This was supported by subgroup analysis indicating participants who had started a specific immunosuppressant (mycophenolate mofetil, MMF) within 1 year and had overall better outcomes did not have any indication of modified symptoms with lenabasum, whereas those not taking immunosuppressive treatment or on MMF for more than 1 year had numerically improved scores with lenabasum 20 mg twice-a-day versus placebo. These findings may call for more restricted background immunosuppressive treatment, or more robust subgroup analysis, in future trials to aid clarity in elucidating the potential benefit of lenabasum in SSc [358].

Another phase 3 trial for lenabasum was conducted in patients with dermatomyositis, a chronic autoimmune disease involving skin inflammation, muscle weakness, lung disease and inflammatory arthritis. While “total improvement score” primary and secondary endpoints were not met, improvement versus placebo was observed in two indications, muscle strength and rash, in subgroups separated by the presence of muscle weakness [357]. In addition, biomarker results showed a reduction in the expression of IFN-γ, a cytokine that can induce a pro-inflammatory response in macrophages, even though lenabasum did not significantly alter the number or types of immune cells infiltrating into the biopsied affected skin [356]. An ex vivo investigation on leukocytes from patients in the trial found that both CB2 and PPARγ were involved in responsiveness to lenabasum [386]. Interestingly, monocyte-derived dendritic cells, intermediate monocytes, and CD4+ T cells exhibited a CB2-dependent lenabasum response, whereas other cell types had a co-dependent CB2/PPARγ or solely PPARγ-dependent mechanism. Higher CB2 expression was also correlated with responsiveness to lenabasum, raising the potential for baseline CB2 expression testing to assist in predicting ideal lenabasum candidates (though the same analysis was not undertaken for PPARγ). These observations support the important role of CB2 in the lenabasum mechanism of action. Considering indications of positive outcomes in clinical trials to date, including from a 3-year open label extension to a phase 2 dermatomyositis trial [387], it is hoped that lenabasum may serve as a new approach for treating various inflammatory disorders.

Olorinab (APD371), a CB2-selective full agonist, is under development as a treatment for IBD [388]. As well as high CB2 > CB1 selectivity (over 1000-fold by binding and signalling assays) and weak or no binding to a panel of potential off-targets, it has been designed to have improved drug-like properties in comparison with traditionally highly lipophilic cannabinoids and had low CNS penetration in rats [359]. These properties lend olorinab to potential use in non-CNS conditions such as IBD. In a phase 2a study, two doses of olorinab (25 mg and 100 mg) were evaluated in 14 patients with IBD for 8 weeks [360]. The open-label, randomised trial found improvements in abdominal pain scores from baseline with both olorinab doses, without CNS-related adverse effects. While the study demonstrated promising results, the study was limited by factors such as small sample size and open-label design. This was followed by a phase 2b placebo-controlled trial in patients with irritable bowel syndrome (IBS) [361]. A total of 273 participants were randomised to three olorinab doses or placebo three-times-daily for 12 weeks. Although in overall analysis there was no difference in abdominal pain score from baseline versus placebo for any dose, a significant improvement versus placebo was found for a participant sub-set with higher baseline pain scores and administered the highest olorinab dose.

## 8. Concluding Remarks and Future Perspectives

**Figure 1:**
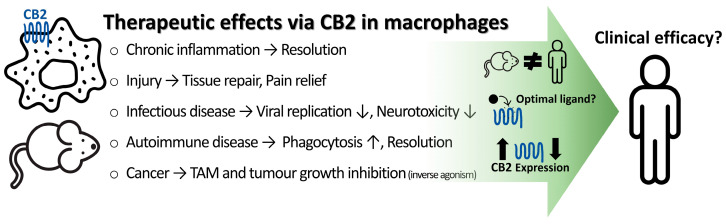
**Therapeutic effects via CB2 in macrophages and the challenges of clinical translation**. Promising therapeutic effects via CB2-mediated regulation of macrophage functions and polarisation have been demonstrated in cell and preclinical animal models in various inflammation-related states and diseases. The next step is to translate these promising preclinical results into clinical efficacy. Key challenges in this translation include that rodent models only partially recapitulate human disease pathology, that CB2-targeted compounds need to be optimised in terms of CB2 selectivity, physiochemistry/pharmacokinetics, and biased signalling properties, and navigating the impact of patient- and disease type/stage-specific factors such as CB2 expression on the opportunity for clinical efficacy. Icons from FreePik (www.freepik.com); figure prepared in Microsoft PowerPoint.

A diverse body of preclinical research indicates that CB2 modulation can provide therapeutic benefits in acute and chronic inflammatory diseases via altering macrophage activity (Figure 1). Although the precise roles and mechanistic understanding of CB2 activation in the immune responses of macrophages are not completely resolved in many contexts, and some applications are better evidenced for the CB2-dependence of effects than others, the overall impression is that of profound opportunity for treating a wide range of conditions. Furthermore, utilising sufficiently CB2-selective compounds eliminates the psychoactive effects induced by CB1 activation or blockade; there has indeed been a lack of adverse effects reported in clinical trials of CB2-targeted compounds to date.

However, despite numerous promising preclinical outcomes, clinical translation of CB2-targeted therapies has not so far been successful. Most CB2-targeted clinical trials have failed to validate therapeutic efficacy, and development of many compounds has been discontinued. Notably, many of the compounds trialled so far have not necessarily been “ideal” candidates in terms of CB2 affinity, selectivity, and physiochemistry [113], and only a narrow range of clinical applications have been trialled relative to the potential utility indicated in preclinical research. Furthermore, efficacy has been reported in some trials in specific patient sub-populations. As a strategy to improve the likelihood of positive clinical outcomes, patient selection and/or optimisation of treatment timing via monitoring biomarkers capable of predicting patient responsiveness and receptiveness to CB2-targeted therapies could be implemented. Potential biomarkers for this purpose include macrophage activation or polarisation markers in blood immune cells or tissue biopsy, or circulating cytokine profiles. Therefore, the apparently limited efficacy observed so far in clinical trials does not preclude the possibility of CB2 ligands ultimately becoming successful treatments.

Aside from expanding the scope of CB2-targeted clinical trials, some specific challenges in translating preclinical to clinical efficacy are evident. Discrepancies in outcomes may largely be attributable to limitations of rodent models in accurately representing human disease, as well as interspecies differences in CB2 expression and downstream signalling pathways. While model translatability poses difficulty in drug development for many targets and contexts, known discrepancies in signalling and regulation of CB2 between humans and rodents may call for specific experimental design to address these differences and improve accuracy in predicting clinical outcomes [115,133].

The complexity of immune-related diseases in human patients also implies the likelihood of diversity in responses and outcomes from treatments in comparison with model organisms which are typically homogenous. A key factor underlying the potential for clinical efficacy of CB2-targeted drugs is the expression level of CB2, which differs depending on the disease type and progression stage, and may be variable between individuals. To address this issue, it may be useful to assess CB2 expression in candidate patients to evaluate suitability for CB2-targeted treatment, for example, via tissue biopsy or with PET ligands (Section 4.2). Moreover, as the CB2 gene has a number of SNPs, including non-synonymous SNPs that may alter receptor function and/or ligand engagement directly, SNP genotyping may be insightful when assessing patient-specific variations in response to CB2-targeted therapies [141].

While we have here specifically reviewed the role of CB2 in macrophages, it is important to keep in mind that CB2 can modulate the effects of other immune cell types, as well as some non-immune tissues, which could potentially have conflicting impacts or feedback effects on macrophage responses. While most whole animal preclinical studies incorporate this aspect by default, lack of interactions between cell types and organ systems may well limit the applicability of reductionist in vitro investigations. The potential to couple CB2 ligands with macrophage-targeting strategies may be interesting to explore.

An exciting opportunity, currently completely unexplored in the context of CB2 modulation of macrophages or in clinical trials, is the development and characterisation of CB2 ligands with distinct functional properties and the potential to hone specific signalling pathways and downstream outcomes. CB2 can activate multiple intracellular signalling pathways, and different ligands can preferentially trigger distinct signalling cascades in a phenomenon known as biased agonism. Spatial and temporal signalling biases have been reported (see also Section 3.4). These opportunities for regulating signalling may enable the selective amplification of inflammatory regulation and potentially minimise unwanted outcomes. However, little research to date has investigated CB2 biased agonism in the context of human physiological or disease conditions, and the bias profiles of CB2 ligands that have already undergone trial are largely unknown. An additional under-explored opportunity, currently in its infancy but with similar theoretical benefits, is the development of CB2 allosteric modulators.

In summary, preclinical research demonstrates broad therapeutic potential for CB2-mediated control of macrophage-involving immune responses, but this promise has not yet reached fruition in demonstrating clinical efficacy. We anticipate that through the availability of highly CB2-selective ligands, with continued attention to understanding the associated pharmacodynamics, improved consideration to preclinical model translatability, further expansion of clinical trials, and honing clinical trial design, safe and effective immunomodulatory CB2 therapies might not be too far from the horizon.

## Data Availability

No new data were created or analysed in this study. Data sharing is not applicable to this article.

## Abbreviations

The following abbreviations are used in this manuscript:

2-AG	2-arachidonoylglycerol
7KC	7-ketocholesterol
AD	Alzheimer’s Disease
ad lib.	Ad libitum
AEA	Anandamide
AJA	Ajulemic acid
ALS	Amyotrophic lateral sclerosis
ALT	Alanine aminotransferase
APP	Amyloid precursor protein
Arg1	Arginase 1
aq.	Aqua (in water)
ASC	Apoptosis-associated Speck-like protein containing a Caspase recruitment domain
ATP	Adenosine triphosphate
Aβ	AD-associated amyloid β
BBB	Blood–brain barrier
BCP	β-(E/trans)-Caryophyllene
BDNF	Brain-derived neurotrophic factor
BLM	Bleomycin
BMDMs	Bone marrow-derived macrophages
cAMP	Cyclic adenosine monophosphate
CAR-M	Chimeric antigen receptor-macrophage
CATB	Cathepsin B
CB1	Cannabinoid receptor 1
CB2	Cannabinoid receptor 2
CBD	Cannabidiol
CCI	Controlled cortical impact
CD	Crohn’s disease
CF	Cystic fibrosis
CFA	Complete Freund’s adjuvant
APP/PS1	Chimeric mouse/human APP with mutant human presenilin 1
CINV	Chemotherapy-induced nausea and vomiting
CLP	Cecal ligation puncture
CNS	Central nervous system
COX	Cyclooxygenase
CRISS	Combined Response Index in diffuse cutaneous systemic sclerosis
DAMP	Danger-associated molecular pattern
DNFB	1-fluoro-2,4-dinitrobenzene
EAE	Experimental autoimmune encephalomyelitis
EGFR	Epidermal growth factor receptor
EMT	Epithelial-to-mesenchymal transition
EP2	PGE2 receptor 2
ERK	Extracellular signal-regulated kinase
FDA	Food and Drug Administration
fMLP	Formyl-methionyl-leucine-phenylalanine
GABA	γ-aminobutyric acid
GC	Glucocorticoid
GDP	Guanosine diphosphate
GM-CSF	Granulocyte-macrophage colony-stimulating factor
GPCR	G protein-coupled receptor
GRK	GPCR kinase
GRP	Gastrin-releasing peptide
GTP	Guanosine triphosphate
HAND	HIV-associated neurocognitive disorders
HD	Huntington’s disease
HIV-1	Human immunodeficiency virus type 1
HIVE	HIV encephalitis
iBCG	Irradiated *Mycobacterium bovis*-BCG
IBD	Inflammatory bowel disease
IBS	Irritable Bowel Syndrome
ICH	Intracerebral haemorrhage
ICTF	Trifluoro-icaritin
IFN-γ	Interferon-γ
IL	Interleukin
iNOS	Inducible nitric oxide synthase
i.p.	Intraperitoneally
IST	Immunosuppressive treatment
JAK/STAT	Janus-activated kinases-signal transducer and activator of transcription
JNK	C-Jun N-terminal kinase
KO	Knockout
LPS	Lipopolysaccharide
MAGL	Monoacylglycerol lipase
MAPK	Mitogen-activated protein kinase
MCP-1	Monocyte chemoattractant protein-1, CCL2
M-CSF	Macrophage colony-stimulating factor
MDM	Monocyte-derived macrophage
MDMA	3,4-Methylenedioxymethamphetamine
MHC	Major histocompatibility complex
MMF	Mycophenolate mofetil
Mrc1	Mannose receptor 1
MS	Multiple sclerosis
MTB	Mycobacterium tuberculosis
NFT	Neurofibrillary tangle
NF-κB	Nuclear Factor kappa B
NK	Natural killer
NLR	NOD-like receptor
NO	Nitric oxide
NRF2	Nuclear factor erythroid 2-related factor
oxLDL	Oxidised LDL
PAMP	Pathogen-associated molecular pattern
PBMC	Peripheral blood mononuclear cells
PD	Parkinson’s disease
PEA	Palmitoylethanolamide
PERK	Protein kinase R-like endoplasmic reticulum kinase
PGE2	Prostaglandin E2
PI3K	Phosphoinositide 3-kinase
PMA	Phorbol-12-myristate-13-acetate
p.o.	Orally
PPAR	Peroxisome proliferator–activated receptor
PRR	Pattern recognition receptor
PVR	Proliferative vitreoretinopathy
QA	Quinolinic acid
RA	Rheumatoid arthritis
RANTES	Regulated on activation, normal T-cell expressed and secreted, CCL5
ROS	Reactive oxygen species
SBP	Spontaneous bacterial peritonitis
SIM	Spontaneously immortalised microglia
SLE	Systemic lupus erythematosus
SNP	Single nucleotide polymorphism
SOCS3	Suppressor of cytokine signalling 3
SR1	SR141716A
SR2	SR144528
SSc	Systemic sclerosis
TAM	Tumour-associated macrophage
Tat	Trans-activating protein
TBI	Traumatic brain injury
TCA	Tricarboxylic acid
TGF-β	Transforming growth factor-β
Th1	Type 1 T helper
THC	Δ9-tetrahydrocannabinol
TLR	Toll-like receptor
TMEV	Theiler’s murine encephalomyelitis virus
TNF	Tumour necrosis factor
TRPV1	Transient receptor potential vanilloid 1
UC	Ulcerative colitis
VEGF	Vascular endothelial growth factor
